# Cultural group selection and human cooperation: a conceptual and empirical review

**DOI:** 10.1017/ehs.2020.2

**Published:** 2020-02-07

**Authors:** Daniel Smith

**Affiliations:** Bristol Medical School, Population Health Sciences, University of Bristol, Bristol BS8 2BN, UK

**Keywords:** Cultural group selection, cooperation, culture, norm psychology, institutions, altruism

## Abstract

Cultural group selection has been proposed as an explanation for humans’ highly cooperative nature. This theory argues that social learning mechanisms, combined with rewards and punishment, can stabilise any group behaviour, cooperative or not. Equilibrium selection can then operate, resulting in cooperative groups outcompeting less-cooperative groups. This process may explain the widespread cooperation between non-kin observed in humans, which is sometimes claimed to be altruistic. This review explores the assumptions of cultural group selection to assess whether it provides a convincing explanation for human cooperation. Although competition between cultural groups certainly occurs, it is unclear whether this process depends on specific social learning mechanisms (e.g. conformism) or a norm psychology (to indiscriminately punish norm-violators) to stabilise groups at different equilibria as proposed by existing cultural group selection models. Rather than unquestioningly adopt group norms and institutions, individuals and groups appear to evaluate, design and shape them for self-interested reasons (where possible). As individual fitness is frequently tied to group fitness, this often coincides with constructing group-beneficial norms and institutions, especially when groups are in conflict. While culture is a vital component underlying our species’ success, the extent to which current conceptions of cultural group selection reflect human cooperative evolution remains unclear.

## Graphical abstract

**Figure:**
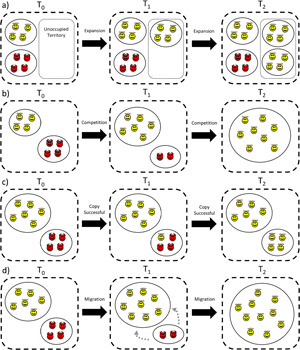

**Media Summary:** Culture is a key human adaptation, but if and how Cultural Group Selection has shaped human cooperation remains unclear.

## Introduction

Compared with other species, humans are unmistakably outliers. We can live in practically any environment, reside in groups numbering millions of people and cooperate extensively with unrelated individuals we will never meet again. This behaviour appears difficult to reconcile with a strict Darwinian fitness-maximising perspective. Despite this rather anomalous behaviour, humans are undoubtedly the most successful species on Earth. While our success has historically been attributed to various factors, it is now commonly agreed-upon that our capacities for culture and cooperation are the twin pillars underlying our success (Boyd 2017; Henrich 2015; Laland 2017; Pagel 2012). Despite this agreement, there is currently little consensus regarding the pathways by which these remarkable capacities evolved and how culture and cooperation interact to produce our status as biological outliers.

One approach which attempts to answer these questions is ‘cultural group selection’ (hereafter CGS). Despite growing in popularity over the past few decades, there has been much debate surrounding CGS. While previous papers have explored many of the themes discussed below, these publications have tended to focus on either: (a) broader issues of using a cultural evolutionary approach to understanding human cooperation (e.g. André and Morin 2011; El Mouden *et al*. 2014; West *et al*. 2011), rather than CGS in particular; or (b) critiquing specific aspects or predictions of CGS (e.g. Lamba 2014; Lamba and Mace 2011; and responses to Richerson *et al*. 2016), rather than a broader overview of CGS. CGS is a fraught and keenly debated topic; it is hoped that this review will provide some clarity regarding how CGS operates, its relation to ‘altruism’ in humans, and whether CGS provides a convincing explanation for human cooperation.

This paper is structured as follows. First, we begin with a brief discussion of the historical roots of CGS by discussing group selection theory more generally and its relation to kin selection as frameworks for understanding cooperative evolution. CGS models are then introduced. Following this, several assumptions underlying CGS are evaluated against existing evidence. The concluding section offers a brief attempt at synthesis to bridge the gap between CGS and traditional models of cooperative evolution, as well as outstanding questions for future research.

## Historical roots of cultural group selection: genetic group selection

Prior to the 1960s, the level at which natural selection was believed to operate was often unclear. Some believed that selection acted on individuals; others advocated that selection acted on groups, while others – such as Darwin in *The Descent of Man* (Darwin 1871) – used a combination of both (for a historical account, see Sober and Wilson 1998).

However, since Hamilton (1964) proposed his theory of ‘inclusive fitness’, the possibility of selection acting solely at the group level became less tenable (see also Williams 1966; for a glossary of key terms, see Box 1). According to this perspective – later dubbed the ‘kin selection’ framework – organisms are expected to maximise their inclusive fitness, which is the sum of their direct fitness (the effect of their actions on their own lifetime reproductive success) and indirect fitness (the effect of their actions on the lifetime reproductive success of others, weighted by relatedness between partners). This can be summarised by Hamilton's rule, which states that a behaviour will evolve if *rB − C* > 0, where ‘−*C*’ is the direct fitness effect, and ‘*rB*’ is the indirect fitness term, composed of ‘*B*’ (benefit to others) and ‘*r*’ (coefficient of relatedness). Based on this kin selection approach, individually costly group-beneficial behaviours are unlikely to evolve under many conditions. In a population of altruists (who pay a direct fitness cost to help others) and defectors (who pay no costs but benefit when interacting with altruists), if interactions are random (*r* = 0) then strategies of defection spread until no altruistic agents remain (Nowak 2006). In order for altruism to evolve, it must be directed towards relatives who are more likely to share the same trait (i.e. *r* > 0).

Box 1: Glossary

*Inclusive fitness* – the sum of an individual's direct fitness (−*C*) and indirect fitness (*rB*), where ‘*r*’ is the coefficient of relatedness between a focal individual and their partner(s), ‘*B*’ is the benefit to the recipient when the focal individual performs a behaviour, and ‘*C*’ is the cost to the focal individual of performing said behaviour. Both ‘*B*’ and ‘*C*’ are measured in terms of lifetime reproductive success.

*Coefficient of relatedness (r)* – the extent to which interacting individuals share the cooperative trait, beyond the baseline frequency of the trait in the population (i.e. whether there is assortment); *r* = 1 means perfect positive assortment (i.e. altruists only meet other altruists), while *r* = 0 means that interactions are random (i.e. altruists meet other altruists in proportion to their frequency in the population). This definition can apply to both genetic and cultural relatedness (see Sections S1 and S2 of the Supplementary Information, respectively). For genetic traits, most relatedness is due to common ancestry, so ‘*r*’ often approximates the proportion of shared genes over the whole genome (*r* = 0.5 for full siblings, r = 0.125 for cousins, etc.).

*Kin selection* – the approach to social evolution based on decomposing fitness into a direct fitness component (−*C*) and an indirect fitness component (*rB*). As defined by Hamilton's rule, a behaviour can evolve if the indirect fitness benefit (*rB*) to performing a social behaviour, minus the direct fitness cost (*C*) to the focal individual of performing said behaviour, is greater than zero (*rB – C* > 0). Note that kin selection can also be defined more narrowly as a selection process where a behaviour is selected via indirect fitness effects through genetic relatives (i.e. when *rB* > 0), but here I am defining kin selection more broadly as an organising framework for thinking about social evolution based on direct and indirect fitness components.

*Kin selection altruism (KS-altruism)* – from a kin selection perspective, altruism is a behaviour which decreases an individual's lifetime direct fitness (although it may increase their indirect fitness). That is, the ‘*C*’ term in Hamilton's rule is positive (meaning there is a direct fitness cost).

*Multi-level selection (MLS)* – the evolutionary framework based on selection acting both within and between groups. The relative contribution of each level of selection determines what evolves.

**Figure 1.**
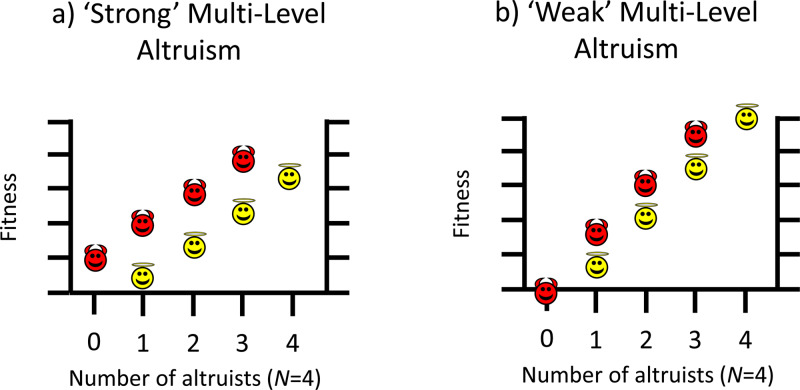
Graphical depiction of ‘weak’ and ‘strong’ varieties of altruism from a multi-level selection perspective. In both cases altruists (angel icons) have lower relative fitness than defectors (devil icons) within groups. (a) *Strong multi-level altruism*: in randomly formed groups, altruists have lower relative and absolute fitness than defectors. In order for strong ML-altruism to evolve, groups must form non-randomly (i.e. altruists assorting with other altruists). (b) *Weak multi-level altruism*: in randomly formed groups, altruists have lower relative fitness than defectors, yet have higher absolute fitness than if said individual was selfish. If the strength of between-group selection is strong enough then weak altruism can evolve, even if groups are formed randomly. Note that strong ML-altruism is altruistic from a kin selection perspective as the direct fitness term in Hamilton's rule is negative (−*C* < 0), while weak ML-altruism is not altruistic from a kin selection perspective as the direct fitness term in Hamilton's rule is positive (*−C* > 0). See Figure 2 for a worked example.

**Figure 2.**
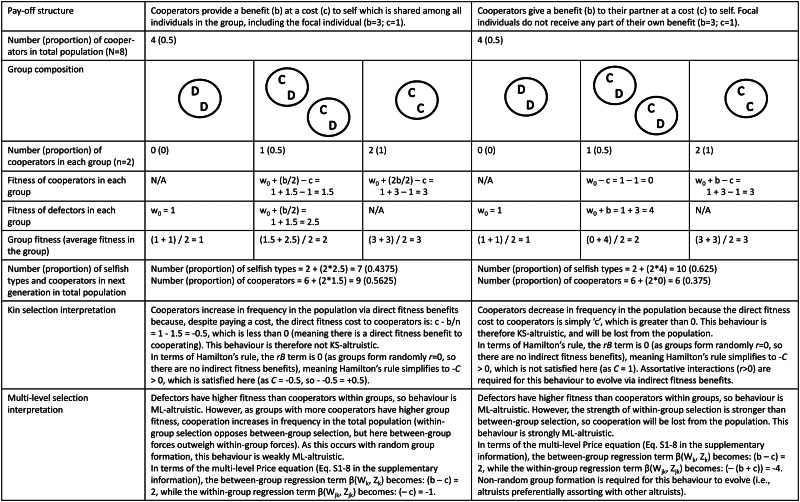
Worked example demonstrating the difference between definitions of ‘altruism’, including altruism as defined from a kin selection perspective, weak multi-level altruism and strong multi-level altruism. In these simple models there are two types of individuals: cooperators (C) and defectors (D). Individuals randomly pair off into groups of two (so *r* = 0), where they interact for one round before reproducing based on their pay-offs (pay-offs differ depending on the pay-off structure; see top row). To avoid situations of negative fitness, all agents begin with one unit of baseline fitness (*w*_0_).

*Multi-level altruism (ML-altruism)* – from a multi-level selection perspective, altruism is a behaviour which is selected against within groups (because altruists have lower fitness than selfish individuals within a group), but favoured in competition between groups (because groups with more altruists have greater fitness than groups with fewer altruists). Note that not all ML-altruistic behavior is KS-altruistic (see Figures 1 and 2).

*Cultural group selection (CGS)* – the process by which populations with group-beneficial cultural traits outcompete other groups. Requires mechanisms to stabilise behaviour, reduce variation within groups and/or reduce or eliminate within-group selection against cooperators (e.g. by social learning strategies and/or a norm psychology), followed by selection acting between groups to select the most cooperative groups.

*Equilibrium selection* – selection between groups at different stable equilibria. Multiple stable equilibria may be reached by processes of cultural transmission, rewards and punishment. Selection then acts on these stable equilibria, favouring more cooperative and cohesive groups.

*Norm psychology* – a collection of psychological mechanisms which motivate individuals to observe group norms and punish norm-violators.

*Norms* – behavioural standards shared by a group. Norms are group-level properties and are characterised by moralistic reactions to violations of such behavioral standards.

*Institutions* – mechanisms to structure social interactions. Institutions change the ‘rules of the game’ and facilitate cooperative behaviour by altering the pay-off structure and therefore the costs and benefits to cooperation and defection. Institutions can involve coordinating social interactions, monitoring others’ behaviour and sanctioning defectors. They range from informal systems (such as gossip, kinship systems and taboos) to formal systems (such as legal systems and police forces).

While this reconceptualisation effectively ended the ‘for the good of the species’ group selection thinking, a new branch of ‘multi-level selection’ (MLS) arose, originally based on the Price equation (Price 1972). This approach decomposes selection into between- and within-group components. If the population is structured into groups, then within-group selection favours non-cooperators (as selfish individuals have greater fitness than altruists within groups), while between-group selection favours altruists (as groups with more altruists have greater fitness than groups with fewer altruists). Therefore, if the strength of between-group selection is strong enough and/or within-group selection weak, then seemingly altruistic traits can spread in the population (Okasha 2006; Sober and Wilson 1998).

The difference between these viewpoints is in the perspective taken, rather than the underlying evolutionary process (Kerr and Godfrey-Smith 2002; West *et al*. 2007). From a kin selection perspective, behaviours which benefit others can spread if individual fitness also depends on group fitness (in which case cooperative individuals increase their direct fitness by helping the group) or if cooperation increases the fitness of relatives (an indirect fitness benefit). From an MLS perspective, group-beneficial behaviours can evolve if between-group selection outweighs within-group selection, with kin-groups one mechanism of population structuring to assort altruists together. Although these two conceptualisations appear rather disparate, they predominantly differ in one crucial respect: kin selection approaches take an ‘individualist’ view, in which the group is part of an individual's environment, meaning that fitness is only assigned to individuals; whereas MLS approaches take a ‘collectivist’ view, in which group-level fitness is modelled separately from individual-level fitness (where group fitness is simply the average fitness within said group). In many cases it is relatively straightforward to switch from an ‘individualist’ to a ‘collectivist’ perspective, or vice versa (Kerr and Godfrey-Smith 2002).

The extent to which MLS and kin selection models are mathematically equivalent is, however, a contentious topic, with views ranging from complete equivalence (Gardner *et al*. 2011; Marshall 2011) to equivalence only under very limited conditions (Nowak *et al*. 2010; van Veelen *et al*. 2017). To some extent these debates revolve around different conceptions of the various terms in Hamilton's rule and their interpretation, particularly in non-additive systems (Birch 2014, 2017; van Veelen *et al*. 2017). Additivity refers to situations where the costs and benefits to cooperating do not depend on the behaviour of one's partner (e.g. in a classic Prisoner's Dilemma scenario the costs and benefits are identical, regardless of how one's partner acts; see Figure 2 for such an example). Non-additivity, in contrast, refers to situations where the costs and benefits are dependent on the behaviour of one's partner (e.g. the benefits to cooperating may be greater/lower if one's partner also cooperates; Queller 1985). Some authors claim that non-additive situations cannot be modelled using Hamilton's rule – and therefore that kin selection and MLS approaches are not equivalent – as the direct and indirect fitness terms cannot be easily represented in terms of fixed ‘costs’ and ‘benefits’ (as they require additional ‘synergistic’ or other terms; Van Veelen 2011; van Veelen *et al*. 2017). In response, supporters of the equivalence thesis claim that a fully general regression-based method can be used to calculate the direct and indirect fitness terms even in non-additive situations, and therefore that kin selection and MLS are mathematically equivalent approaches (Gardner *et al*. 2011; Marshall 2011). Others disagree, and claim that the direct and indirect fitness terms from this generalised regression-based method lack a natural interpretation (as in non-additive situations ‘*B*’ and ‘*C*’ are not fully under the actor's control, but rather also depend on the population structure; Allen *et al*. 2013; Nowak *et al*. 2017), and that this regression-based method can lead to model misspecification in non-additive systems (van Veelen *et al*. 2017). However, additive models do not suffer from these complications, and can be easily analysed from both perspectives (van Veelen *et al*. 2017; see Section S1 of the Supplementary Information for an additive model which can be interpreted easily from both perspectives, as well as additional discussion on this topic). The simple models presented in Figure 2 also assume additive pay-offs, as this is a useful place to start to explore how these different perspectives map on to one another and how they differ. The extent to which this MLS–kin selection equivalence holds more generally is beyond the scope of this paper and does not directly impact the discussion below regarding cultural group selection.

As a consequence of these different perspectives, kin selection and MLS define some key terms differently – such as ‘altruism’ – which may foster confusion between perspectives. Kin selection defines altruism as behaviour that lowers an individual's direct fitness (i.e. the ‘*C*’ term in Hamilton's rule is positive), so can only evolve via indirect fitness benefits through kin (Hamilton 1964; West *et al*. 2007). In contrast, MLS defines altruism solely in terms of within-group selection (i.e. behaviour which decreases fitness within a group), regardless of whether it benefits the individual in between-group competition (Kerr *et al*. 2004; Sober and Wilson 1998; Wilson and Wilson 2007).

Altruism from an MLS perspective may either be ‘weak’ or ‘strong’ (Wilson 1990). In both cases the within-group selection term (equation S1–7 in the Supplementary Information) is negative, indicating that such behaviour is selected against within groups. However, the conditions under which weak and strong ML-altruism can evolve are different. Strong ML-altruism occurs when altruistic individuals have lower fitness than defectors within groups and lower fitness than if they were not altruistic (Figure 1a). Weak ML-altruism occurs when altruists have lower fitness than defectors within groups, yet higher fitness than if they were not altruistic (Figure 1b). Importantly, weak ML-altruism can evolve if groups are formed randomly (i.e. *r* = 0), while strong ML-altruism can only evolve if there is non-random group formation (i.e. *r* > 0), with altruists preferentially assorting together (Wilson 1990). Put another way, in weak ML-altruism individuals increase their direct fitness by cooperating (the ‘−*C*’ term in Hamilton's rule is positive), while for strong altruism individuals decrease their direct fitness by cooperating (the ‘−*C*’ term in Hamilton's rule is negative). As such, weak ML-altruism is not altruistic from a kin selection perspective, even though the within-group selection term is negative. However, strong ML-altruism *is* equivalent to KS-altruism, and hence both require assortative interactions to evolve (Hamilton 1975).

To avoid potential confusion, I will use the terms KS (kin selection)-altruism and weak or strong ML (multi-level)-altruism to distinguish between these different definitions. For a worked example of the difference between KS-altruism, weak ML-altruism and strong ML-altruism, see Figure 2. This is an oversimplified example to help fix ideas; in more complex cases, especially those involving non-additive payoffs, group conflict, population replacement and strong selection pressures, it can be difficult to calculate the direct and indirect benefits necessary for a kin selection analysis from certain MLS models (Lehmann and Keller 2006). This issue is particularly apparent in models of cultural evolution which often adopt an MLS perspective, as these models are often framed in terms of group competition and rapid cultural adaptation, which may be more difficult to model from a kin selection perspective (Boyd 2017, p.108; Boyd *et al*. 2011).

It is important to recognise that these different definitions of altruism do not make different predictions regarding evolutionary change in terms of gene frequencies (or of cultural traits), but rather they differ in the interpretation of said behaviour. Take the first example in Figure 2, where individuals receive some pay-off from their own cooperative behaviour. From a kin selection perspective this behaviour is not KS-altruistic as individuals increase their direct fitness by cooperating, while from an MLS perspective this behaviour is (weakly) ML-altruistic, as within mixed-groups cooperators have lower fitness than defectors. The evolutionary change in gene frequencies is identical (helping behaviour will increase in the population), but the interpretation of whether this behaviour is altruistic differs depending on the perspective taken.

To summarise this brief sketch of group selection, I reiterate that: (a) MLS is not an alternative evolutionary process to kin selection, the two views only differ in perspective, and; (b) MLS and kin selection have different definitions of altruism which should not be conflated. Additionally, while kin selection and MLS are equally valid perspectives for thinking about evolutionary change, thus allowing pluralism in approaches, some questions are easier to understand from an ‘individualist’ kin selection perspective, while others are easier to understand from a ‘collectivist’ MLS perspective; neither view is ‘correct’ at the expense of the other, but one may lead to a more intuitive understanding for certain problems (Kerr and Godfrey-Smith 2002). This paper does not therefore endorse one view over the other, but rather aims to describe how these perspectives link together and where this can lead – and has led – to conflicting views and debate. Now, I turn to cultural group selection and the evolution of human cooperation more specifically.

## Cultural group selection

**Figure 3.**
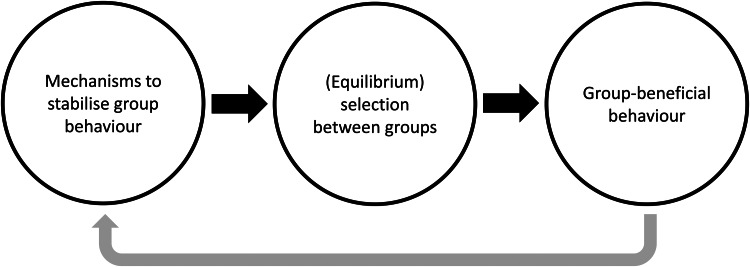
Simplified schema showing the process of cultural group selection. The grey arrow indicates that the process of cultural group selection can further select for proximate mechanisms which facilitate subsequent cultural group selection.

Cultural group selection aims to understand the remarkable levels of human cooperative diversity through the interplay between the dual inheritance systems of genes and culture (Boyd and Richerson 1985). It explicitly takes an MLS approach (Boyd and Richerson 2009a; Henrich 2004a; Richerson *et al*. 2016) and proposes that group-beneficial behaviour can evolve via cultural processes which stabilise behaviour and reduce variation within groups, followed by selection acting between these groups (see simplified schema in Figure 3). In the absence of cultural transmission, genetic group selection is unlikely to play a major role in human evolution given high levels of observed migration; any altruistic group can be invaded by selfish individuals, and therefore proliferate and remove the required genetic differences between groups necessary for group selection. However, cultural traits are independent of genes, so between-group differences can withstand migration if mechanisms to stabilise group behaviour exist. This may include social learning biases, such as conformism or prestige-biased transmission (Boyd and Richerson 1985), or punishment of those who violate shared behavioural standards, a so-called ‘norm psychology’ (Chudek and Henrich 2011). With behaviour stabilised and variation reduced, the scope for between-group selection to outweigh within-group selection is greater, allowing group-beneficial behaviour to evolve under a wider range of conditions.

Many CGS models rely on ‘equilibrium selection’. Via the stochastic processes of reward, punishment and social learning, different groups reach different stable equilibria: some groups will be replete with cooperators, others full of non-cooperators. Given this arrangement, selection between groups can then operate, leading to the differential success of more cooperative groups as they outcompete less-cohesive communities (Boyd and Richerson 2009a; Henrich 2004a; Richerson *et al*. 2016). This may then further select for proximate mechanisms, such as conformism, norm-internalisation or cooperative preferences, which further act to stabilise group behaviour and promote cooperation (the grey arrow in Figure 3). As a result of this iterative gene-culture co-evolutionary process, it is frequently claimed that humans evolved other-regarding preferences, meaning that individuals may often act against individual self-interest (Bowles and Gintis 2011). Examples include cooperating with strangers in one-shot interactions (Ensminger and Henrich 2014) and punishing norm-violators simply because it is normative (Boyd 2017). Models of cultural group selection can be derived which are equivalent to the genetic models in Section S1 of the Supplementary Information, but based on cultural rather than genetic inheritance (Section S2 of the Supplementary Information). The question of whether CGS acts on cultural or biological fitness is returned to in Section 8. With this background in mind, I now aim to explore the assumptions underlying CGS, the evidence in support of these claims and some of the areas of conceptual ambiguity in current formulations of CGS.

## The assumptions and theoretical foundations of cultural group selection

### Does cultural group selection make unambiguous predictions about human behaviour?

There are some core elements of CGS that make it an evolutionary process which plausibly explains elements of human cooperation (Richerson *et al*. 2016): (a) cultural variation is responsible for some behavioural differences between groups; (b) certain cultural traits are inherited and persist over time; and (c) different cultural traits are responsible for the success of certain cultural groups. In some cases the evidence for CGS is irrefutable: larger and more organised groups with better technology have outcompeted and displaced smaller and less organised groups with simpler technology throughout human history (Currie and Mace 2009; Diamond 1997). Examples include agriculture replacing hunting-and-gathering, the colonisation of the Americas and the rise of nation states. Despite this broad high-level agreement, in discussions and models of CGS there are different varieties of CGS which can result in this process and need to be separated. These often make different predictions regarding human behaviour and altruism.

Some presentations of CGS claim that it provides an explanation for altruism in humans (Henrich 2004a; Richerson and Boyd 2005). These explanations focus on mechanisms of social learning, such as conformism or prestige-bias, which have evolved because they are adaptive in most contexts but may be maladaptively applied when acquiring cooperative behaviour. Taking an example from Richerson and Boyd (2005, pp. 162–163), consider two groups linked by migration: one group is predominantly religious and the other is non-religious. In the non-religious group, no-one helps anyone. In the religious group, individuals altruistically help one another. Within each group, migrants adopt the prevailing behaviour via conformism. However, individuals also have a preference to adopt the selfish non-religious behaviour, meaning that in the religious group there will always be a proportion of non-religious free-riders who have greater fitness than religious individuals. Thus, being religious is both KS- and ML-altruistic, as individuals would have greater fitness if they were non-religious. However, owing to conformism these group differences can persist, and religious groups will outcompete non-religious groups in between-group competition (for a formal model of this process, see chapter 7 of Boyd and Richerson 1985). Richerson and Boyd (2005) label such behaviours ‘maladaptive adaptations’, since they rely on constraints on social learning strategies which can lead to maladaptive behaviour. As such, I will call these explanations ‘maladaptive CGS’ (while noting that, on average, these strategies may be adaptive for acquiring traits other than cooperation).

Maladaptive CGS was invoked in many early discussions of CGS. As Richerson and Boyd (2005) state, ‘if cultural rules arise [via conformism] that cause individuals to sacrifice their own interests for the good of the group, group selection can cause the frequency of individually costly but group-beneficial traits to increase’ (p. 162), and that CGS is an ‘engine for generating maladaptations from the narrow genes’-eye point of view’ (p. 244). Note that not all models of CGS based on social learning result in maladaptive behaviour; pay-off-biased imitation – where individuals preferentially copy fitness-enhancing traits – can also lead to the spread of cooperative behaviour, either by imitating the behaviour of successful groups (Boyd and Richerson 2002) or by migrating to successful groups and then adopting the local behaviour (Boyd and Richerson 2009b). However, when applied within groups, pay-off-biased transmission will frequently not lead to stable cooperative outcomes, as defectors have greater fitness than cooperators within groups, so pay-off-biased transmission will lead to these selfish behaviours being copied (van den Berg, Molleman, and Weissing 2015).

Rather than focus on social learning mechanisms to explain cooperative behaviour, other, often more recent, discussions of CGS tend to focus on mechanisms of reward, reputation, punishment and a norm psychology; what I will dub ‘normative CGS’. From this perspective, behaviour is not altruistic as cooperators would not have lower fitness than defectors within groups (Boyd 2017; Boyd and Richerson 2009a). Take punishment: if all individuals in a group punish norm-breakers (and punish those who do not punish norm-breakers), punishment can stabilise any behaviour within groups (Boyd and Richerson 1992). As non-punishers would have lower fitness, this behaviour is not maladaptive or altruistic by either kin selection or MLS definitions. As Boyd (2017) recently stated, CGS ‘is about selection among groups with different social arrangements that are evolutionarily stable, *not* about the evolution of individually costly group-beneficial behavior’ (p. 107; emphasis in original) and that ‘cultural group selection predicts that norms maintained by self-interest will tend to be group beneficial, not that people will be altruists’ (p. 189). However, other authors do claim that such punishment of norm-breakers *is* against self-interest, and therefore altruistic (Bowles and Gintis 2011). Note that normative and maladaptive CGS can work in concert, such as a conformist bias to punish norm-breakers (Henrich and Boyd 2001), but I largely treat them as separate processes for conceptual clarity and because they make qualitatively different predictions regarding human cooperative behaviour.

Rather than focus on mechanisms to stabilise group behaviour followed by equilibrium selection, other formulations of CGS emphasise the conflict between within- and between-group selection, without necessarily requiring stable group behaviour (Sober and Wilson 1998; Waring *et al*. 2015), which I will call ‘mechanism-neutral CGS’. For instance, Turchin *et al*. (2013) and Smaldino (2014) define CGS (what they call ‘cultural multi-level selection’) as the process by which complex and otherwise-costly group-beneficial cultural institutions can proliferate over other institutions owing to between-group competition, especially warfare (by ‘costly’, I mean that the within-group term is negative, but the trait is maintained via its benefits in between-group competition; it does not imply that the trait is KS-altruistic). From this mechanism-neutral CGS perspective, social learning mechanisms and/or a norm psychology to stabilise group behaviour are not necessary as the proximate mechanisms are left unspecified. Rather, costly institutions are maintained because of inter-group competition; without group competition, these institutions would probably become simpler and less costly. Although mechanism-neutral CGS is similar to maladaptive and normative CGS in many respects, in that all require group competition for group-beneficial behaviour to spread, a crucial difference is that the latter do not require group competition for cooperation to be sustained, as cooperation is stable within groups. As mechanism-neutral CGS does not rely on mechanisms to stabilise between-group differences, group competition is required to maintain costly institutions (this also means that groups need not be in equilibrium, and therefore that mechanism-neutral CGS does not rely on equilibrium selection). Mechanism-neutral CGS may be weakly ML-altruistic, but it does not require KS-altruism, strong ML-altruism or that behaviour is maladaptive. Unless otherwise specified, when mentioning ‘CGS’ below I am referring to maladaptive and normative CGS, rather than mechanism-neutral CGS.

All varieties of CGS presented above agree that competition between cultural groups is important, but they differ in the proximate mechanisms and processes by which this is achieved and whether the resultant behaviour is altruistic or self-interested (Table 1). These conflicting definitions and differing behavioural expectations can make testing prediction of CGS difficult. Many critiques of CGS focus on maladaptive CGS and argue that content-blind mechanisms of social learning (e.g. conformism) are unlikely to explain human cooperation (e.g. Lamba and Mace 2011). This conclusion is likely to be true. However, as CGS does not only rely on blind social learning mechanisms, the dismissal of CGS based on a rejection of maladaptive CGS may be rather uncharitable. At the same time, a proliferation of theories which fall under the name ‘cultural group selection’, yet make different predictions regarding human cooperative behaviour, is almost certain to foster confusion.

**Table 1.** Summary of predictions and proposed proximate mechanisms made by different versions of cultural group selection (CGS).

Type of cultural group selection (example reference)	Maladaptive CGS (Boyd and Richerson 1985)	Normative CGS (Boyd 2017)	Mechanism-neutral CGS (Turchin *et al*. 2013)
Group competition required to spread cooperative norms/institutions	Yes	Yes	Yes
Key proximate mechanism(s) to maintain group differences	Social learning (conformism and prestige-bias)	Norm psychology to punish norm violators	Unspecified
Requires stable norms	Yes	Yes	No
Costly behaviours maintained in the absence of group competition	Yes	Yes	No
Conflict between within- and between-group selection	Yes (?)^a^	No	Yes
Behaviour may be KS-altruistic or maladaptive	Yes	No (yes according to some)	No

^a^ In the religious vs non-religious group example presented in the main text, there is conflict between the levels of selection, as in the religious group the minority of non-religious individuals has greater within-group fitness than the religious individuals. Despite within-group selection acting against religious individuals, conformism can still maintain between-group variation.

### Is altruism defined consistently?

In addition to ambiguity over whether CGS results in altruistic behaviour, the literature also contains multiple definitions of key terms such as altruism (Deb and Smith 2019). One key terminological difference already discussed is between KS-altruism and ML-altruism (either weak or strong; Figure 1). When discussing potentially altruistic behaviour in humans, some authors define altruism from a kin selection perspective (André and Morin 2011; El Mouden *et al*. 2014; West *et al*. 2007), others from an MLS perspective (Bowles and Gintis 2011; Sober and Wilson 1998). However, other authors working on human cooperative evolution define altruism as short-term costs (Fehr and Fischbacher 2003; Gintis 2000), even if in the long-term it may increase lifetime biological fitness, such as via reciprocity, reputation or success in group conflict.

These conflicting definitions of altruism lead to different assessments of whether cooperation is altruistic or not. For instance, MLS and short-term perspectives define altruism more broadly than kin selection. This may be one of the reasons why many authors who adopt either of these perspectives emphasise that altruism among non-relatives is rife in humans (Bowles and Gintis 2011; Henrich *et al*. 2005; Sober and Wilson 1998), while authors who adopt a kin selection view claim that cooperation towards non-kin is mutually beneficial, not altruistic (West *et al*. 2011, 2007). Some of the controversy surrounding CGS and human cooperation may be due to different definitions of ‘altruism’, with much of the CGS literature adopting altruism as defined by MLS or short-term costs (e.g. Bowles and Gintis 2011; Henrich 2004a; Sober and Wilson 1998), and other evolutionary researchers adopting the KS-altruism definition. However, as discussed above, in more recent CGS publications there has been a shift away from interpreting behaviour as altruistic, as punishment makes otherwise-costly cooperation self-interested (Boyd 2017). As with the differing expectations of behaviour based on maladaptive, normative and mechanism-neutral CGS described above, multiple definitions of terms such as ‘altruism’ can make it difficult to explicitly operationalise and test predictions of CGS.

## Examining the assumptions of maladaptive CGS

In this section I assess the evidence for maladaptive CGS as an explanation for human cooperation, focusing on the social learning mechanisms by which individuals acquire cooperative behaviour.

### Is social learning naïve?

A central premise of maladaptive CGS models is that humans naively apply cultural learning strategies, such as conformism or prestige-biased transmission, to acquire behaviour, with little concern for individual pay-offs (Boyd and Richerson 1985; Henrich 2004a; Richerson and Boyd 2005). These mechanisms are ‘blind’, in that individuals use these strategies even if they lead to maladaptive behaviour. While this may occur in some instances – evolved adaptations are never completely infallible – if these maladaptive social learning strategies persisted for long enough then it is likely that they would be outcompeted by other more sophisticated strategies (El Mouden *et al*. 2014). Relying on maladaptive social learning mechanisms to explain human cooperative behaviour is equivalent to a ‘mismatch’ between evolved cognitive mechanisms in ancestral vs modern environments; strategies such as conformism and prestige-bias may have been adaptive in our evolutionary past, but misfire and result in maladaptive behaviour in novel situations (André and Morin 2011; Mace 2011). Alternatively, these occasionally maladaptive strategies may persist owing to cognitive constraints. While a strategy may be adaptive on average, it may misfire in certain situations (such as regarding cooperation), and the costs to investing in a more discriminating strategy – say, a ‘conform for foraging techniques, but not cooperation’ rule – is not offset by the gains (Boyd and Richerson 1985). However, as detailed below, human social learning strategies are incredibly variable and context-specific, and often give rise to adaptive behaviour, even in novel environments, giving less plausibility to both mismatch and constraint-based arguments.

To be adaptive, social learning must be strategic and context-specific (André and Morin 2011; Cronk 2017; Laland 2004). Non-human animals flexibly use complex social learning strategies, including ‘when’ strategies, such as ‘copy when uncertain’ or ‘copy when asocial learning is costly’, and often use multiple social learning strategies in conjunction (Laland 2004). These strategies can lead to adaptive behaviour, such as stickleback fish using flexible pay-off-based social learning to optimally exploit resources (Kendal *et al*. 2009), or great tits using a combination of conformism and individual reinforcement when learning to acquire adaptive foraging techniques (Aplin *et al*. 2017). Given our reliance on culture one would expect the social learning strategies employed by humans to be even more discerning and context-dependent (Cronk 2017). Indeed, humans deploy several different adaptive social learning strategies depending on the situation (Morgan *et al*. 2012). In economic games with the opportunity for social learning, individuals tend to use these strategies flexibly in order to maximise their earnings; that is, social learning was motivated by income maximisation, such as adopting a ‘copy-the-successful’ strategy, which in turn often reduced cooperation as the economically successful individuals were the most selfish (Burton-Chellew *et al*. 2015, 2017). Although experiments have shown that individuals do use frequency-based information in social dilemmas, this is distinct from conformism as it is not neutral with regard to pay-offs and does not reduce variation within groups (van den Berg *et al*. 2015). Thus, cultural learning mechanisms which stabilise and reduce group differences, such as conformism, are not frequently observed in experiments, especially in cooperative situations (Efferson *et al*. 2007; Eriksson and Coultas 2009; Lamba 2014). Laland has summarised this succinctly; ‘Human beings copy; they copy a great deal. But they do not slavishly copy. Slavish copying would not be adaptive’ (Laland 2017, p.64). This is not to say that culturally acquired behaviour will always be adaptive. Clearly social learning rules can be misapplied and result in inefficient or maladaptive behaviour (Laland and Williams 1998; Richerson and Boyd 2005), but where cultural traits impact biological fitness and culture causes maladaptive behaviour, there will be selection pressure in favour of alternative strategies which enhance biological fitness (El Mouden *et al*. 2014).

### Do humans learn cooperative behaviour culturally?

For CGS to occur, cultural mechanisms must stabilise and reduce within-group variation in behaviour. Several aspects of human behaviour are indisputably cultural as they could not have originated within one generation; think complex technology (Henrich 2004b), institutional complexity (Currie *et al*. 2010) and religion (Watts *et al*. 2015). Problems occur, however, in applying the same logic to individual-level behaviours such as cooperation, which can emerge via several pathways, including fixed genetic behaviours, phenotypic plasticity, epigenetic inheritance, individual learning and social learning (or combinations thereof; Jablonka and Lamb 2014). Unlike complex technology which is absent outside humans, many other species – including bacteria (Kümmerli *et al*. 2009) – display variability and plasticity in cooperative behaviour (Adams *et al*. 2015). As cooperation can evolve in the absence of social learning, this means that there are multiple proximate pathways by which cooperative behaviour can evolve. Many of these make similar predictions regarding the distribution of cooperative behaviour, making them difficult to differentiate. For instance, both phenotypic plasticity and conformism predict that, given different environments, behaviour within groups will be more similar than behaviour between groups, irrespective of genetic differences.

It is often assumed that differences in cooperative behaviour are socially learned, such as by conformism (Boyd and Richerson 1985), copying cooperative prestigious individuals (Henrich *et al*. 2015) or pay-off-biased imitation (Boyd and Richerson 2002). While individuals do appear to learn some cooperative behaviour via social learning, when it is employed it tends to be based on pay-off-biased transmission (Burton-Chellew *et al*. 2017), rather than conformism (Lamba 2014; little empirical work has explored whether adults learn cooperative behaviour via prestige-bias; see Henrich *et al*. 2015). Individuals also readily adapt their behaviour via individual evaluations of the situation: for instance, in the absence of social learning individuals alter their cooperative behaviour in consistently adaptive directions according to cues of anonymity (Ernest-Jones *et al*. 2011), whether competition is within or between groups (Puurtinen and Mappes 2009), and whether interactions are repeated (Rand and Nowak 2013). Furthermore, individuals vary their evaluations of resource distributions based on local ecological factors such as the role of luck in acquiring resources, social homogeneity, warfare and resource availability (Nettle and Saxe 2019). This variation can be explained by individual-level adaptive decision-making, without the need for acquiring cooperative behaviour culturally. This provides an alternative explanation for cross-cultural differences in cooperation (e.g. Henrich *et al*. 2005), without ascribing these differences to cultural learning. This is not to say that cultural transmission is unimportant for acquiring cooperative behaviour, but at present this is largely an untested assumption and probably occurs in combination with other processes (Lamba 2014). However, the evidence for widespread conformism – a key mechanism for stabilising group behaviour – is rather weak.

## Examining the assumptions of normative CGS

This lack of empirical support for widespread conformism, coupled with the ability of individuals to alter cooperation in adaptive ways in the absence of social learning, suggests that maladaptive CGS is unlikely to explain large-scale human cooperation. In the following section I focus on normative CGS, based on the punishment of norm-breakers, to maintain group differences, and discuss whether this provides a more plausible mechanism for group-level variation in cooperation.

### Do norms determine behaviour?

Normative CGS proposes that individuals acquire arbitrary group norms of cooperation owing to a norm psychology, where individuals adopt the norms of the local group and punish norm-violators. Norms are ‘learned behavioural standards shared […] by a community’ (Chudek and Henrich 2011, p. 218). It is important to differentiate this normative definition of ‘norm’ from descriptions of actual behaviour – that is, distinguishing between how individuals *ought* to act and how they actually *do* act (Wallen and Romulo 2017). As norms are by definition group-level traits, one would expect low levels of within-group variation in stated norms, beliefs and values, as appears to be the case (Bell *et al*. 2009; although see Eriksson and Coultas 2009). However, as actual behaviour, not norms or beliefs, drives evolution, the extent to which norms translate into behaviour is a key question.

**Figure 4.**
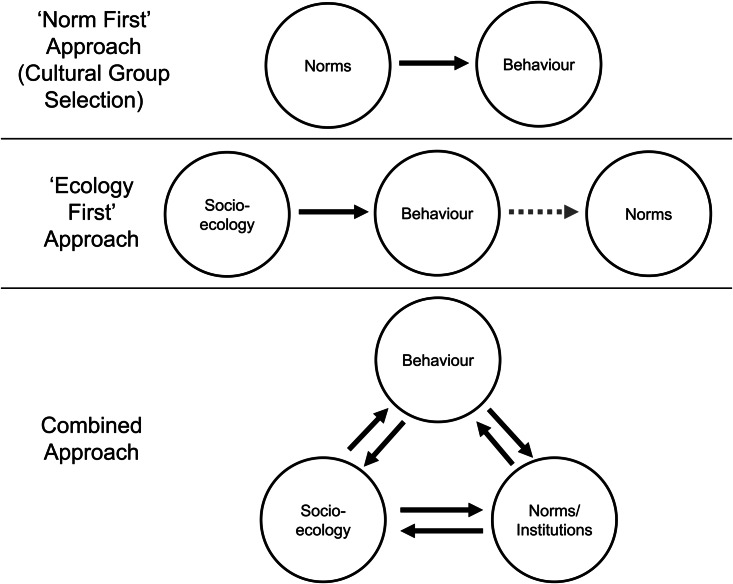
Determinants of cooperative behaviour from a cultural group selection ‘norm first’ perspective (upper), a socioecological ‘ecology first’ approach (middle) and a combined approach (bottom). ‘Socioecology’ is defined as the social, economic and physical environment, so includes subsistence patterns, demography/group size, group competition, sedentarisation, etc., as well as culturally evolved behaviours (e.g. residence patterns, technology). ‘Behaviour’ is how individuals actually behave, ‘norms’ are shared beliefs about the ‘correct’ behaviour in a group, while ‘institutions’ are structures which shape social interactions and alter the pay-offs to cooperation and defection. *Cultural group selection* (upper): cultural group selection is generally silent about the role of socioecology and tends to portray cooperative behaviour as solely determined by norms. *Socioecological approach* (middle): socioecology (broadly defined) determines behaviour, which in turn may influence shared norms (the shaded arrow from behaviour to norms). *Combined approach* (bottom): both the socioecology and culturally evolved norms/institutions impact behaviour, with behaviour also evaluating and updating existing norms/institutions (where possible) and shaping socioecological circumstances (e.g. by niche construction). Norms and institutions therefore also impact socioecology in path-dependent ways, while current socioecological circumstances – which may be norm/institution-dependent – shape and constrain future norms and institutions. Thus, norms/institutions and socioecology feed into one another and may be difficult to separate in practice (see Box 2). This combined approach involving reciprocal feedback loops is likely to be necessary in explaining large-scale cooperation.

Normative CGS requires that cultural norms cause cooperation (Figure 4, upper). In some cases the association between norms and behaviour is strong, such as Maasai *osotua* needs-based cooperation (Cronk 2007), food-sharing among Samburu pastoralists (Lesorogol 2007) and reciprocity among Saami reindeer herders (Thomas *et al*. 2018). Elsewhere, there are discrepancies between norms and behaviour. As predicted by genetic evolutionary models, Mukogodo parents preferentially invest in daughters over sons as they live in relative poverty compared with their neighbours, so males are less able to compete in the mating market. However, there is a mismatch as the stated Mukogodo norm is to prefer sons, a norm probably borrowed from their higher-status Maa-speaking neighbours (Cronk 2017). Tibetan pastoralists display the same mismatch: norms which prefer sons, yet daughter-biased parental investment is observed (Du and Mace 2017). If the norms individuals espouse are divorced from behaviour, then norms cannot always shape individual actions.

Furthermore, a correlation between norms and cooperation is not necessarily evidence that norms cause behaviour. In many cases behaviour is shaped by the socioecology, which in turn shapes norms (Figure 4, middle). For instance, among Chinese farmers historical ecological differences in subsistence practices (rice- vs wheat-farming) are associated with present-day behavioural and psychological differences, as rice-farming requires greater cooperation and interdependence than wheat-farming (Talhelm *et al*. 2014; although for a re-evaluation of this claim see Roberts 2015). Similarly, within-society variation in cooperation – where society-level norms are relatively constant – can be explained by differences in socioecology, such as demography, need and social networks (Lamba and Mace 2011; Smith *et al*. 2016). This ‘ecology first’ approach (as opposed to the CGS ‘norm first’ approach) can also explain cases where behaviour may be adaptive but the norms are outdated (Du and Mace 2017), owing to cultural lag.

### Why follow norms and punish norm-breakers?

Behaviour determining norms cannot explain all aspects of human cooperation. Many examples presented above are individual choices (such as sex-biased parental investment) or small-scale cooperation (such as forager food-sharing) which plausibly do not require norms in order to evolve (although these behaviours may be enshrined in norms after their original evolution). Large-scale cooperative ventures, such as warfare and common pool resource management (resource extraction, irrigation systems, fishing rights, etc.) are more difficult to evolve, given that the scope for cooperation declines with group size (Powers and Lehmann 2017). CGS may provide a plausible explanation for these large-scale cooperative ventures.

While maladaptive CGS can lead to altruistic behaviour, normative CGS is generally considered consistent with self-interest. That is, when cultural norms are backed up by punishment of norm-violators, it is in an individual's self-interest to cooperate and follow group norms (Boyd 2017; Boyd and Richerson 2009a; Chudek and Henrich 2011; Zefferman and Mathew 2015). The famous ‘second-order free-rider problem’ comes into play here; even though everyone is better off by cooperating and punishing free-riders, individuals who free-ride on others’ altruistic punishment will have greater fitness than punishers, causing the population to evolve towards non-cooperation. In small groups or situations of strong between-group competition, this second-order free-rider problem may not arise as individuals may increase their direct fitness by punishing, even though others in the same group will have higher fitness (assuming groups are composed of non-kin, in these cases the behaviour would be weakly ML-altruistic, not strongly ML-altruistic or KS-altruistic; Lehmann *et al*. 2007). However, in larger groups individuals are unlikely to gain direct fitness benefits by cooperating, even with group competition present. This occurs because in large groups punishment is unlikely to have a significant impact on group success, so the direct fitness gains are minimal and unlikely to outweigh the costs (Boyd 2017; Powers and Lehmann 2017).

A potential solution to this problem is that cooperation can be stable if punishing norm-violators is itself normative (Boyd 2017; Boyd and Richerson 1992). That is, individuals punish norm-breakers simply because they are violating a norm. Competition between groups can then act to select the most cooperative equilibria. The question then becomes: why is punishing norm-violators normative? One proposed answer is based on an evolved norm psychology which arose in the context of small groups where there were direct benefits to punishing norm-breakers. With this psychological machinery in place, it could then be harnessed via CGS for large-scale cooperative projects (Boyd 2017, pp. 118–120).

Normative CGS provides a plausible theory for large-scale human cooperation and makes several predictions which can be tested empirically, some of which are not unique to CGS (e.g. reputation-based theories also predict that humans should take an interest in third-party behaviour). One unique prediction is that individuals should indiscriminately punish norm-breakers simply because they broke a norm, and not for any direct fitness benefit (other than to avoid potential higher-order punishment for not punishing).

Despite theoretical coherence, the evidence that individuals indiscriminately punish norm-violators is rather weak. Some experiments have shown that individuals punish others at a cost to self, even in one-shot interactions (Bernhard *et al*. 2006; Fehr and Fischbacher 2004; Henrich *et al*. 2005). This behaviour is *prima facie* support for a norm psychology, and is often claimed to be altruistic (Bowles and Gintis 2011; Boyd *et al*. 2003). However, in repeated situations these seemingly altruistic individuals increase levels of cooperation, often resulting in greater pay-offs for the group and for themselves (dos Santos *et al*. 2013; Gürerk *et al*. 2006). These ‘altruistic punishers’ also receive other direct benefits, such as a reputation for trustworthiness (Jordan *et al*. 2016) or other reputational or monetary incentives (Ostrom 1990). Experiments which have pitted predictions of normative CGS (in which punitive behaviours are indiscriminately directed towards norm-violators) against predictions from a self-interested model (in which punishment brings greater future cooperation to the punisher) have found that behaviour conformed to the latter. This suggests that individuals do not punish defectors solely to maintain the group norm, but rather punish selectively to encourage cooperation and increase future pay-offs for themselves (Krasnow *et al*. 2012; see also Burton-Chellew and West 2013; Pedersen *et al*. 2018). Thus, while punishment of norm-breakers certainly does occur (Mathew and Boyd 2011; Wiessner 2005), in contrast to predictions made by normative CGS, this punishment appears self-interested (beyond avoiding higher-level punishment).

## On the origins of norms and institutions

Although both conformist and prestige-biased social learning strategies and a norm psychology are plausible mechanisms for explaining group differences in cooperation on which equilibrium selection can act, the evidence in support of these mechanisms is far from conclusive. This poses a problem for CGS, as for large-scale cooperation to evolve there must be mechanism(s) to sustain such behaviour. In this section I assess the evidence regarding how group differences arise and whether these processes lead to stable differences in cooperation, and explore a potential alternative hypothesis for the evolution of large-scale cooperation based on institutional evolution (Powers and Lehmann 2013; Powers *et al*. 2016). Institutions are mechanisms to structure social interactions which alter the pay-offs to cooperating and defecting (North 1991; Ostrom 1990). By manipulating the ‘rules of the game’, individuals and societies can create institutions which facilitate large-scale cooperation, such as via mechanisms to monitor peoples’ behaviour and reputation as future social partners, rules to coordinate interactions, or structures which incentivise punishment. This institutional approach may overcome many of the limitations of maladaptive and normative CGS discussed above, in addition to other issues regarding the origin and stability of group-beneficial behaviours discussed below.

### Do group differences arise via stochastic processes and are norms stable?

For CGS to occur, there must be differences between groups. In maladaptive CGS the origin of groups at different cooperative equilibria is not formally modelled, but rather is assumed to occur via stochastic processes (Boyd *et al*. 2011). In normative CGS, groups vary randomly as a result of punishment and reward processes which reach different stable equilibria; some groups develop cooperative norms, others do not (Boyd 2017; Ensminger and Henrich 2014). As stated by Boyd and Richerson (2009a, p. 3273) ‘[t]he problem is that the three Rs [reputation, reciprocation and retribution] can stabilise *any* behaviour. If everybody agrees that individuals must do *X*, and punish those who do not do *X*, then *X* will be evolutionarily stable as long as the costs of being punished exceed the costs of doing *X*. It is irrelevant whether *X* benefits the group or is socially destructive. It will pay to do *X*’. Although maladaptive and normative CGS processes posit different proximate mechanisms, they agree that different groups will reach different equilibria, after which equilibrium selection can act.

While these are plausible mechanisms to stabilise group-level behaviours, theories of institutional change based on individuals or groups creating cooperative institutions provide an alternative explanation which may be better supported by empirical evidence. Maladaptive and normative CGS predict that behaviour within groups is stable, so even if superior equilibria exist it will be difficult for groups to change their norms endogenously. However, studies on institutional change indicate that individuals are often aware that they are in a poorly functioning group and frequently try and create new rules to enhance fitness (Ostrom 1990; Singh *et al*. 2017). For instance, in the 1970s the Turkish fishing village of Alanya was suffering from an over-exploitation of fishing stocks. After a decade of trial-and-error, the village settled on a new system for governing fishing rights which benefits everyone and permits cheaters to be detected and punished swiftly (Ostrom 1990, pp. 18–20). This institutional change arose based on rational self-interest, without requiring conformism/prestige-bias, a norm psychology or group competition. The change was also endogenous, rather than copying the institutions of more successful groups, so cannot be attributed to CGS.

‘Rational-actor’ theories of institutional change can explain how individuals cooperate in large-scale projects via self-interest, without relying on altruistic cooperation, maladaptive social learning strategies or a norm psychology (Powers *et al*. 2019). One way institutions solve the second-order free-rider problem is by rewarding punishment, thereby aligning punishment with self-interest. Examples include punishers keeping the fines taken from defectors, as found in many common-pool resource systems worldwide (Ostrom 1990). If cooperative reputations are known and the exclusion of individuals with poor reputations is possible, then institutions which ostracise defectors from partaking in subsequent mutually beneficial interactions can evolve which also avoid the second-order free-rider problem (Greif 1993; Panchanathan and Boyd 2004). Each of these processes results in the within-group selection term in the multi-level Price equation being positive, meaning that cooperators have greater fitness than defectors within groups (or, from a kin selection view, the individuals increase their direct fitness by cooperating). Put another way, individual-level processes – not just competition between groups – can lead to the spread of cooperative behaviour (Chaudhary *et al*. 2016). Formal models of voluntary investment in group-beneficial institutions have indicated that this process can result in stable systems of costly institutions and can evolve from an asocial state (Powers and Lehmann 2013). This also means that that stochastic processes are not required to explain the origins of between-group variation, as required by models of maladaptive and normative CGS.

This institution-based account is not necessarily a wholesale alternative to CGS. If groups are in competition then both CGS and institutional accounts predict that more-cooperative groups will prosper at the expense of less-cooperative groups (Richerson *et al*. 2016). The difference between these theories occurs in the mechanisms which give rise to between-group variation in cooperative behaviour. Normative CGS relies on a norm psychology to punish norm-violators, while maladaptive CGS relies on social learning mechanisms to stabilise group behaviour. Although the specific psychological mechanisms are not modelled, institutional approaches rely on individuals negotiating rules to promote cooperation, coordinate behaviour, punish defectors and reward those who punish free-riders. In this institutional view the second- (or third-, or fourth-, or *n*th-) order free-rider problem is not solved by the punishment of norm-violators being normative or by content-blind social learning mechanisms, but rather by rewards, monitoring mechanisms and incentives to coordinate behaviour and align punishing with self-interest.

Institutional accounts also diverge with predictions from CGS in another respect. In CGS, behaviour is stable within groups, meaning that group competition is necessary to select the more cooperative groups (Boyd 2017; Ensminger and Henrich 2014). In contrast, institutional accounts do not necessarily require group competition in order for groups to become more cooperative, as this process can occur endogenously (Powers and Lehmann 2013; although in other circumstances group competition may be required to uphold otherwise-costly institutions, see Turchin *et al*. 2013). Group competition may of course facilitate the spread of group-beneficial institutions, but group conflict is not a necessary condition for these institutions to evolve (in this respect, when considering group competition institutional accounts can be thought of as examples of mechanism-neutral CGS: see Table 1).

From cross-sectional data it is difficult to distinguish between these alternative theories, as all predict that group-beneficial behaviour should be relatively common. Longitudinal and historical data are important sources required to determine the processes which lead to the emergence of institutions. While current evidence is rather fragmentary, several case studies, such as fishing rights in Alanya, demonstrate how individuals negotiate group-beneficial norms and institutions within groups (Ostrom 1990; Singh *et al*. 2017). Ostrom provides a detailed example of institutional change in sustainable groundwater basin extraction in California which arose endogenously and without group competition (see Ostrom 1990, chapter 4; although surrounding districts later borrowed and adapted these institutions, which could be considered CGS). Experiments have also demonstrated how communication and punishment can lead to the emergence of cooperative institutions in the absence of group conflict (Ostrom 2006). Additionally, although many cultural traits possess a phylogenetic signal, suggesting long-term cultural stability (Mathew and Perreault 2015), other traits relating to cooperation, such as food-sharing, show little phylogenetic signal (Ringen *et al*. 2019). Studies on forager cooperation have shown that cooperative behaviour is variable within ethnographic groups (Smith *et al*. 2018), often based on differences in ecology (Lamba and Mace 2011; Smith *et al*. 2016), and changeable within the space of a single generation (Gurven *et al*. 2002). These findings are consistent with this lack of phylogenetic signal and suggest that cooperative behaviour can display a rapid adaptive response to changing environments. This rapid endogenous adaptive change is difficult to reconcile with CGS, as the time taken for cultural traits to spread via inter-group conflict is likely to be upwards of 500 years (Soltis *et al*. 1995: although other CGS processes of selective migration and copying successful groups can occur on faster time-scales, they are unlikely to explain these patterns of short-term adaptive variation).

A further prediction of CGS which can be tested is norm content. Maladaptive and normative CGS predict that mechanisms to stabilise behaviour should lead to groups at multiple different equilibria, many of which include neutral or positively harmful traits (Ensminger and Henrich 2014). Many cultural traits are certainly puzzling from a purely biological fitness-maximising perspective (Edgerton 1992), but whether they actually damage fitness is harder to assess. Many seemingly harmful cultural practices may have an adaptive basis, such as lethal warfare (Chagnon 1988), female genital cutting (Howard and Gibson 2017) and witchcraft accusations (Mace *et al*. 2018), while for other traits if their effects on fitness are negligible then they could have plausibly evolved by cultural drift (El Mouden *et al*. 2014). In a survey of 60 cultures, the behaviours considered ‘moral’ were strikingly uniform across all societies and are predicted by evolutionary models (Curry *et al*. 2019). These include helping kin, helping the group, reciprocity and bravery. This lack of cross-cultural variation argues against the CGS idea that norms can stabilise any behaviour. Rather, the behaviours which are normative tend to be broadly fitness-enhancing. Of course, perhaps all of the poorly adapted groups with anti-social norms have already been replaced, but the studies above detailing how new norms and institutions arise suggest that they are generally tailored to be both group- and individually beneficial, rather than owing primarily to stochastic processes.

While additional data is required, the current evidence argues against CGS, which suggests that group-level differences in cooperation are largely based on social learning strategies and/or a norm psychology to stabilise any cooperative behaviour. Instead, based on the current technological, social, ecological, institutional and economic constraints, individuals and groups try to design norms and institutions which benefit themselves; as individual fitness is frequently tied to group fitness, norms and institutions are likely to be broadly group-beneficial. Norms and institutions are continually being evaluated, contested and (re-)shaped (Morin 2016a; Ostrom 1990; Singh *et al*. 2017), arguing against the stability of norms. There will still probably be selection between different norms/institutions, as some will be better-suited than others in a given ecology, but this process does not necessarily require norm-compliance and randomly formed group-level stable strategies, as proposed by maladaptive and normative theories of CGS.

## On the spread of group-beneficial norms

In this section, I explore the process(es) by which group-beneficial norms, institutions and behaviours spread via CGS. This involves discussing the difference between selection acting on biological vs cultural fitness, as well as whether CGS is a group-level process. I also discuss whether CGS is an alternative to approaches based on biological fitness.

### Does cultural group selection act on cultural fitness, biological fitness, or both?

Although CGS may impact genetic evolution and biological fitness, primarily it is concerned with the spread of cultural traits. As stated by Richerson *et al*. (2016, p. 5), ‘[a] cultural variant that attracts many imitators has a reproductive success which is not necessarily tied to biological reproduction. [In the case of CGS] we can use models to specify the costs, benefits, and success of cultural variants in terms of changes in their frequency’. This cultural fitness perspective can be expressed using a cultural multi-level Price equation (see Section S2 of the Supplementary Information). However, a focus solely on cultural fitness does not necessarily equate to an impact on biological fitness.

A key distinction is between cultural traits which impact biological fitness (reproductive success or inclusive fitness) and cultural traits which impact cultural fitness (the cultural influence of an individual/group for a specific trait). Birch (2017) refers to these as Cultural Selection 1 (CS1) and Cultural Selection 2 (CS2), respectively. CGS always acts on CS2 (the cultural trait's impact on cultural fitness) but does not always act on CS1 (the cultural trait's impact on biological fitness). Take competition between economic organisations (Richerson *et al*. 2016). Certain businesses have greater cultural fitness than others, in terms of either survival or being imitated. This can be thought of as CGS acting via cultural fitness (CS2). However, the impact of being part of a successful business on biological fitness (CS1) may be minimal, given that employees from unsuccessful organisations are unlikely to have lower reproductive success (they can just get jobs elsewhere). In this example CS1 and CS2 are decoupled, so although more successful businesses have greater cultural fitness, this has little impact on biological fitness.

In other situations, cultural and biological fitness are more tightly linked. Warfare may be a good example. Success in warfare frequently depends on cultural factors such as social organisation and technological innovations. Groups possessing these traits are able to out-compete less-organised and less technologically complex groups (Diamond 1997; Turchin *et al*. 2013), meaning that being part of such groups may enhance both biological and cultural fitness. However, vanquished individuals from losing groups are often assimilated within the winning group, rather than killed outright (Richerson and Boyd 2005; Soltis *et al*. 1995). Cultural and biological fitness will be completely aligned if individuals from losing groups are eliminated, while if warfare is largely symbolic with few casualties and individuals from losing groups assimilated within the victorious group, then selection acts predominantly on cultural fitness, not biological fitness (the cultural spread of cooperative groups may favour subsequent selection on genes for cooperativeness – see grey arrow in Figure 3 – but this selection is distal, rather than immediate).

In practice it is often difficult to distinguish between these processes. Consider the Nuer/Dinka conflict – frequently presented as a canonical case of CGS (Richerson and Boyd 2005) – where the superior organisation of the Nuer in inter-group conflict led to them expanding at the Dinka's expense. Although some Dinka were killed, many Dinka assimilated into the Nuer population. A similar process combining both replacement and assimilation has probably occurred throughout human history. Although these processes appear similar when adopting a cultural fitness perspective (CS2) – as the focus is on the spread of the cultural trait – they are different when adopting a biological fitness perspective (CS1); group expansion and conquest without assimilation mean that losing individuals have lower biological fitness, while assimilation does not necessarily entail a decrease in biological fitness to individuals from the losing group. The distinction between cultural traits acting on cultural fitness (CS2) and cultural traits acting on biological fitness (CS1) may be a useful framework for thinking about CGS and whether one is interested in the spread of cultural traits themselves or on the immediate impact of these cultural traits on biological fitness.

### Is cultural group selection a group-level process?

**Figure 5.**
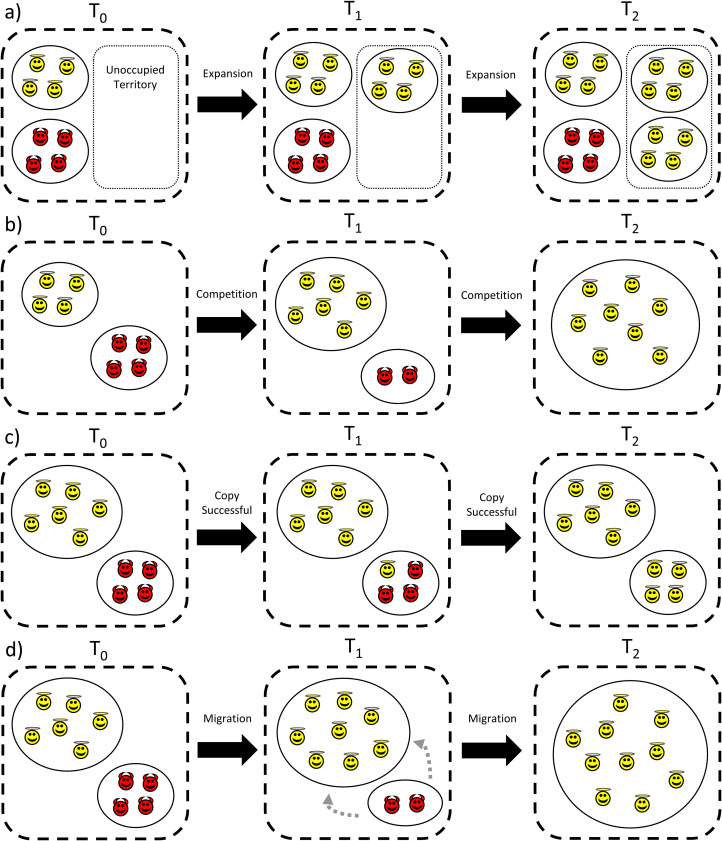
Different cultural group selection processes which result in the spread of group-beneficial norms and behaviours. Angel icons represent cooperators and devil icons represent defectors. (a) *Differential expansion rates*: in the absence of conflict, cooperative groups may expand into unoccupied territory at a faster rate than less-cooperative groups. (b) *Inter-group conflict*: if there is competition between groups, then more-cooperative and cohesive groups will expand at the expense of less-cooperative groups. (c) *Copying successful groups*: individuals from less-successful groups copy the cooperative traits of successful groups. (d) *Differential migration*: individuals from less-successful groups migrate to more-successful groups and adopt their traits. From a biological fitness perspective, processes (a) and (b) may be examples of a selection process acting on groups, while in processes (c) and (d) no selection on biological fitness is occurring as these are choices made by individuals or groups to ostensibly increase future biological fitness. From a cultural fitness perspective, all could be group selection processes acting on cultural fitness (as cooperative groups are more likely to propagate).

Related to the point above, CGS proposes several mechanisms by which group-beneficial traits can spread (Henrich 2004a, 2015; Richerson *et al*. 2016; Richerson and Boyd 2005), which are summarised in Figure 5. Some of these mechanisms are group-level processes which act on populations, such as one group outcompeting another, either directly (via warfare or conquest) or indirectly (via higher reproductive rates, leading to faster diffusion rates into unoccupied environments). As discussed above, in these processes biological fitness (CS1) and cultural fitness (CS2) may be aligned, in that more cooperative and organised groups possess greater cultural and biological fitness (although at other times group competition may spread just cultural traits, with little impact on biological fitness). These are mechanisms of natural selection – either cultural or biological – acting on groups. However, other proposed CGS processes are decision-making mechanisms of cultural transmission. One of these processes is individuals copying successful traits of other groups (Boyd and Richerson 2002), while another is selective migration to successful groups (Boyd and Richerson 2009b). While this could be viewed as cultural selection – as some traits are preferentially imitated over others, meaning they have higher cultural fitness – from the perspective of biological fitness no selection is occurring.

Depending on whether one adopts a cultural fitness or biological fitness perspective, these processes have different interpretations. In terms of cultural fitness, copying traits from successful groups and selective migration may be group-level processes (and hence CGS), as groups possessing certain cultural traits are more likely to be imitated. However, from a biological fitness perspective, these processes are choices by individuals or groups ostensibly attempting to maximise their future biological fitness. While both of these demonstrate how beneficial cultural traits can spread, there is no need to invoke group-level selection processes acting on biological fitness here as individuals (or groups) either adopt, or migrate then adopt, the practices of more successful groups (Amir *et al*. 2016; Morin 2016b).

### Is cultural group selection an alternative to approaches based on biological fitness?

Some proponents of CGS appear to view it as an alternative to existing approaches based on biological fitness. In a recent review, Richerson *et al*. (2016, p. 3) claim that ‘[t]he issue here is not whether effects such as nepotism, reputation building, and other mechanisms of cooperation supported by reciprocity and inclusive fitness exist […] but whether they are sufficient to explain the large-scale cooperation in human societies’. However, when the focus is on biological (rather than cultural) fitness, cultural and biological approaches are difficult to separate, as cultural adaptations often work via mechanisms of reputation, reciprocity, kinship and punishment to enhance biological fitness (Henrich and Boyd 2016; Henrich and Henrich 2007). Witchcraft accusations (Mace *et al*. 2018) and storytelling (Smith *et al*. 2017) are clear examples of cultural factors which impact reputation and shape patterns of cooperation. Kinship norms may also be culturally evolved institutions to coordinate behaviour and facilitate cooperation (Cronk *et al*. 2019). For instance, Lamaleran kinship is based on patrilineages which coordinate collaborative whaling ventures by determining who can work together, thereby avoiding conflict (Nolin 2011). In online marketplaces, institutions facilitate large-scale cooperation via reviewer systems to broadcast sellers’ (and buyers’) reputation, upon which individuals can base future mutually beneficial interactions (Livingston 2005).

While these norms and institutions are certainly products of cultural evolution, whether they evolved via CGS (as opposed to arising endogenously) is unclear. Even if some did evolve by CGS (for instance, online marketplace companies with reviewer systems outcompeting companies without reviewer systems), this is not necessarily in conflict with traditional evolutionary explanations, as these successful institutions harness these kinship, reciprocal, reputational or sanctioning mechanisms more effectively to facilitate large-scale cooperation (Henrich and Boyd 2016). If CGS leads to maladaptive or altruistic behaviour – as claimed by some (Bowles and Gintis 2011; Richerson and Boyd 2005) – then it would be an alternative to these traditional evolutionary explanations. However, much culturally mediated cooperation appears broadly consistent with biological self-interest. Rather, CGS provides a potential answer to how different societies arrive at different norms and institutions which harness these traditional evolutionary explanations for large-scale cooperation. However, knowing that CGS occurred tells one nothing about the mechanism(s) by which one group out-competed another. When considering biological fitness, CGS is simply multi-level selection with a focus on culture as a proximate mechanism by which individuals and groups acquire adaptive behaviour (for additional discussion of the status of culture as a proximate mechanism, see Box 2). CGS does diverge from traditional evolutionary accounts when the focus is on cultural, rather than biological, fitness, but this is because traditional evolutionary accounts which focus on ultimate biological fitness outcomes tend not to be concerned with cultural fitness and the proximate psychological mechanisms underpinning decision-making and cultural transmission.

Box 2: Is culture a proximate or ultimate explanation?

The claim that culture is a proximate mechanism is often met by the retort that culture is an ultimate explanation because cultural history is important. Cultural history is obviously important in determining and constraining current behaviour, as is phylogenetic history in the rest of the natural world. However, studies which claim to show that culture is more important than the environment in determining behaviour (Guglielmino *et al*. 1995; Mathew and Perreault 2015) probably underplay the adaptive nature of culture for various reasons. First, these studies lump all of the environment together, rather than making specific socioecological predictions regarding adaptive behaviour. As discussed in the main text, norms of food-sharing, which probably have a strong impact on individual fitness and are highly dependent on local ecological conditions, show little phylogenetic signal (Ringen *et al*. 2019). In contrast, other cultural traits which have little impact on individual fitness (such as the language spoken) and are not as dependent on the local environment (such as supernatural beliefs) or opaque cultural practices (such as complex technologies) may follow phylogenetic lines of descent more faithfully. Second, cultural mechanisms and ecological adaptations are difficult to separate in practice (Mace, 2014), and both reciprocally feed into one another (Figure 4, lower). For instance, it is often difficult to say whether a subsistence method is a result of cultural history or due to the environment, as the answer is generally a combination of the two (Gavin *et al*. 2018). Humans are certainly cultural animals, but our cultural history becomes part of the socioecology we develop in and adapt to. By the process of niche construction (Laland *et al*. 2001), humans can adapt their behaviour to new environments while maintaining cultural differences, hence why behavioural diversity can persist, even in identical environments (Eugster *et al*., 2017). For instance, agricultural and hunter-gatherer populations frequently inhabit similar environments, yet, in part because of their cultural history, farmers still farm and foragers still forage. This echoes a point raised by William Irons 40 years ago (1979b, pp. 36–37): ‘[p]ast behaviour and culture are translated into present behaviour and culture by sentient, intelligent creatures who evaluate past events in order to choose how to try to influence future ones. […] The information at hand in a particular society at a particular point in time limits possibilities for change in response to environmental change.’

Part of the controversy surrounding whether culture is an ultimate explanation or proximate mechanism probably stems from multiple, mutually exclusive, definitions of these terms. To some, ultimate explanations are defined in terms of whether a behaviour is biologically adaptive, while proximate mechanisms explain how this behaviour works (Scott-Phillips *et al*. 2011). From this perspective, culture is clearly a proximate mechanism which often – but not always – results in fitness-enhancing behaviour. Other authors, however, equate ultimate explanations with ‘evolutionary history’ and proximate mechanisms with ‘development’ (Laland *et al*. 2011; Mesoudi, 2015). According to the latter view, culture is an ultimate explanation as it is an inherited trait and therefore explains why human populations differ, blurring the lines between evolutionary history and development.

Clearly, these two classifications define ‘ultimate’ explanations differently. A more-encompassing distinction, which differentiates these conflicting viewpoints, is based on Tinbergen's (1963) four questions. For each trait, this scheme asks two questions for *why* a trait exists – one based on *function* (is a trait adaptive?) and the other based on *phylogeny* (what is the trait's phylogenetic history?) – as well as two questions regarding *how* a trait exists, one based on *mechanism* (how does a trait work?) and the other based on *ontogeny* (how does a trait develop?). For many human traits, ‘culture’ provides a potential answer to both ‘how’ questions (ontogeny and mechanism), as well as one of the ‘why’ questions (phylogeny), but not to whether the trait enhances biological fitness (function; the other ‘why’ question). Tinbergen's four-way scheme may be a more parsimonious way to interpret culturally evolved behaviours, rather than the dichotomous proximate–ultimate distinction, which blurs evolutionary history and adaptive function.

## Summary and future directions

This paper has aimed to review the theory underlying CGS and the evidence in support of it. In one sense, CGS is almost undeniably true; some groups have expanded over others as a result of cultural factors, such as their social system and technology. However, in another sense current conceptions of CGS do not provide a satisfactory or alternative explanation for human cooperation. These reasons and areas of disagreement are summarised in Table 2.

**Table 2.** Areas of disagreement or ambiguity surrounding cultural group selection (CGS) as an explanation for human cooperation.

Issue	Consequence	Potential solution
Multiple versions of CGS, including different mechanisms to stabilise group behaviour (Table 1).	Difficult to test predictions of CGS, as expected behaviour varies by the version of CGS adopted (e.g. maladaptive CGS predicts altruism, normative CGS does not).	Additional clarity between these versions of CGS and clear predictions to test them.
Multiple definitions of ‘altruism’ (Figures 1 and 2), including definitions based on kin selection, multi-level selection, and short-term costs.	Difficult to link with wider social evolutionary theory, and a lack of clarity over whether CGS-evolved behaviour is altruistic from a life-time biological fitness perspective or not.	Additional clarity when discussing these key terms (e.g. to avoid conflating short-term altruism for reduced life-time fitness).
Mechanisms to stabilise group behaviour – e.g. social learning strategies such as conformism, or a norm psychology – are not well supported by the empirical evidence.	If these mechanisms are not widely employed, alternative explanations must be sought to explain cross-cultural variation in cooperative norms, institutions and behaviour.	Greater focus on how individuals and groups adopt locally adaptive behaviour, such as by creating group-beneficial institutions.
Group differences do not arise via stochastic processes, are not stable through time, and change endogenously.	Rather than unquestioningly adopt norms, individuals and groups design rules, or shape existing ones, to suit their current situation (e.g. forager food-sharing). CGS may still occur, but group behaviour is not necessarily stable through time.	Further studies exploring how groups adapt existing norms/institutions (or create new ones), and how this impacts the potential scope of CGS.
Ecology often shapes cooperation and can explain some cross-cultural variation in behaviour.	CGS may not be required to explain these patterns of cooperation. Norms, institutions and cultural learning are probably important in acquiring cooperation, but whether these norms and institutions evolved by CGS is not always clear.	A greater integration between models of CGS and their interaction with the local ecology to produce adaptive behaviour.
Whether CGS acts on cultural or biological fitness.	CGS is predominantly concerned with the spread of cultural traits, which does not always coincide with a focus on biological fitness, e.g., in all CGS mechanisms in Figure 5 selection acts on cultural fitness, but only group competition and expansion are situations where selection may also act on biological fitness.	Greater recognition of cultural variation acting on cultural fitness vs cultural variation acting on biological fitness, and keeping these processes distinct.
Is CGS an alternative to traditional evolutionary approaches (based on genetic relatedness, reciprocity, reputation, punishment, etc.)?	To the extent that CGS acts on biological fitness, it is not an alternative explanation. Rather, individuals and groups possessing culturally evolved traits which harness these traditional approaches more effectively have greater biological fitness, so may spread via CGS.	As above, maintaining the distinction between cultural variation acting on cultural fitness vs cultural variation acting on biological fitness is key.

A solution to these problems requires integration between cultural evolutionary approaches (including CGS) which emphasise the importance of cultural history in determining behaviour, and rational-choice theories (‘rational’ does not mean ‘conscious’, however), such as human behavioural ecology, which focus on individuals acquiring adaptive behaviour in different ecologies. These approaches are often contrasted, with evidence for one acting as evidence against the other (Mathew and Perreault 2015). However, these approaches are not necessarily in conflict, with culture a key mechanism to acquire adaptive behaviour (Mace 2014). On the whole, we should therefore expect to see cultural behaviour aligning with what is individually adaptive, albeit with a considerable amount of path-dependence owing to cultural history (El Mouden *et al*. 2014; Irons 1979a; Figure 4, lower). This indeed appears to occur in many cases. In addition to the cases of witchcraft beliefs, storytelling, cultural kinship and online marketplace reputation systems outlined above, other examples include: patrilineal descent systems co-evolving with livestock practices (Holden and Mace 2003); dowry payments as female competition (Gaulin and Boster 1990); subsistence systems and inheritance patterns (Holden *et al*. 2003); plant knowledge and child health (Salali *et al*. 2016); and variation in fertility (Mattison *et al*. 2018). A key step is therefore to move beyond oversimplified dichotomies where human behavioural ecology is perceived to only act based on physical environmental differences with little regard for cultural history (e.g. Mathew and Perreault 2015), and where CGS is characterised as solely owing to conformist social learning without regard for individual pay-offs (e.g. Lamba and Mace 2011). Recent empirical work in humans, both at the macro-scale (Botero *et al*. 2014) and at the micro-scale (Colleran 2016; Mattison *et al*. 2018), is beginning to bridge this gap by integrating how adaptive behaviour can arise based on the interplay of ecology, social learning strategies, individual decision-making and wider historical normative and institutional factors.

A key divide between CGS and human behavioural ecology is that the former largely focuses on the spread of cultural traits while the latter focuses on the impact of behaviour on biological fitness. In this sense, they are talking different languages: knowing a trait has greater cultural fitness does not necessarily give any information about whether the trait is linked to biological fitness. When CGS acts on biological fitness, such as via group competition, unless the behaviour is maladaptive it is not an alternative to human behavioural ecology and traditional evolutionary approaches. Instead, groups possessing culturally evolved traits which enhance reproductive success are more likely to succeed in between-group competition. From a human behavioural ecology perspective, when focusing on cultural fitness CGS is: (a) equivalent to focusing on biological fitness if cultural and biological fitness are perfectly aligned (e.g. if losing sides in war get killed), as selection acts on both cultural and biological fitness simultaneously; (b) a proximate mechanism by which individuals or groups acquire adaptive behaviour (by copying successful groups or moving to successful groups); or (c) a mechanism to spread biologically neutral traits if group competition spreads certain cultural traits which have little or no impact on biological fitness (e.g. competition between business firms).

Depending on how one defines ‘cultural group selection’, this review could be seen as vindicating CGS, or as largely unsupportive of it. If CGS is defined in terms of the process of competition between cultural groups, then this view is broadly supported (with caveats about endogenous cultural change and whether group competition applies to CGS acting on biological, and not just cultural, fitness). However, if CGS is defined more narrowly in terms of both competition between stable cultural groups and the psychology which supports this (that is, maladaptive or normative CGS), then current conceptions of CGS are not well supported. Rather than stable group differences arising from stochastic processes which then require equilibrium selection, group-beneficial norms and institutions appear designed by (relatively) rational actors seemingly to maximise their fitness. This process does not require specific social learning mechanisms, unquestioning punishment of norm-breakers, or competition between groups for group-beneficial behaviour to evolve (although competition between groups can help to promote the evolution of otherwise-costly institutions). Instead, it requires that individuals realise that being part of a cooperative group is better than being part of a disorganised group and design norms and institutions accordingly (or adopt or modifying existing norms). This is easier said than done, as large-scale cooperation projects do often fail (Ostrom 1990), but in principle this rational-actor approach based on institutional evolution can explain large-scale human cooperation without requiring a highly specialised norm psychology or frequently maladaptive social learning strategies.

Despite often being portrayed as competing explanations, there is broad agreement between CGS and traditional evolutionary approaches to understanding human cooperative evolution. For the most part, both sides agree that culture is a key factor underlying human cooperation, and that competition between cultural groups has occurred throughout human evolution. Key questions for future research therefore include: do individuals use conformism or prestige-bias to acquire real-world cooperative behaviour, outside of the laboratory? How do we link cooperative and punitive behaviours to life-time fitness consequences to determine whether they are altruistic? Do norms and institutions emerge by rational choice or by stochastic processes, and how do norms and institutions change within groups? How stable are norms and institutions over time and in response to different environments? And, how do we integrate existing models of CGS with mechanisms to account for patterns of local ecological adaptation?

It is hoped that this review has clarified some of the debates surrounding CGS. While every attempt has been made to present a cogent account of CGS, it is possible that I have made some assertions that advocates of CGS may disagree with. Given the often-conflicting viewpoints surrounding CGS, its relation to altruism and whether it is an alternative account to traditional evolutionary approaches, this is likely to be the case. CGS is a fraught and complex topic; hopefully this paper can lead to more productive dialogue and greater conceptual clarity moving forwards.

## Data Availability

There is no data associated with this submission.

## Supplementary material

For supplementary material accompanying this paper visit http://dx.doi.org/10.1017/ehs.2020.2. click here to view supplementary material
