# Supplementary material for: Cultural group selection and human cooperation: a conceptual and empirical review
Source: Evol Hum Sci. 2020 Feb 7;2:e2. doi: 10.1017/ehs.2020.2 (PMC10427285; doi:10.1017/ehs.2020.2)
Supplement: Supplementary file 1 [file S2513843X2000002Xsup001.zip › CGS_Review_SI_UploadToProof.pdf]

Supplementary Information for 'Cultural Group Selection and Human Cooperation: A Conceptual and Empirical Review' by Daniel Smith

S1: The Mathematical Equivalence of Kin Selection and Multi-Level Selection

In this section I present a simple model of altruism which can be understood from both kin selection and multi-level selection (MLS) perspectives. The model assumes asexual haploid individuals with a single locus which codes either for altruism or selfishness. Pairs of individuals engage in a single interaction per generation. Altruists help others (b) at a cost to self (c) in a Prisoner's Dilemma scenario. If two altruists meet they both receive 'b-c'; if two selfish types meet they both receive nothing; a selfish type meeting an altruist receives 'b', while an altruist meeting a selfish type receives '-c'. Parent-offspring heritability is assumed to be perfect.

We begin with the Price equation, which is a fully general method for modelling evolutionary change from one generation to the next (Frank, 1997; Okasha, 2006; Price, 1972)

$$\bar{w}\Delta\bar{z} = cov(w_i, z_i) + E(w_i \Delta z_i), \qquad \text{(S1-1)}$$

where 'z' is the behavioural trait (z_i=1 for altruists and 0 for selfish types), 'w' is individual fitness and the subscript '*i*' denotes the *i*th individual in the population. Without going into the details of deriving equation S1-1 (see Frank, 1997; Gardner, West, & Wild, 2011; McElreath & Boyd, 2007; Okasha, 2006), this equation says that the average change in the trait from one generation to the next (the '$\Delta\bar{z}$' term on the left-hand side of the equation) is a combination of both the covariance between the trait and fitness (the 'selection' component) and the expected (average) fitness-weighted change in the trait from one generation to the next due to non-selective factors (the 'transmission' component). In genetic evolution, the transmission component is often dropped as it is assumed to be 0 (i.e., meiosis is fair and mutation is unbiased). The '$\bar{w}$' term on the left-hand side of equation S1-1 is the average fitness in the population and does not impact the interpretation of the direction of selection (and if we normalise average fitness to '1' this term disappears).

From this equation we can specify the fitness term 'w_i' as a regression equation

$$w_i = a + bz_p - cz_i + e_i, \qquad \text{(S1-2)}$$

where 'a' is the intercept term for baseline fitness prior to interaction, 'bz_p' is the benefit given to others depending on whether their partner is an altruist or not (the partner's phenotype is given by 'z_p'), 'cz_i' is the cost to self when possessing the altruistic trait, and 'e_i' denotes the residuals. Substituting equation S1-2 into equation S1-1 and dropping the transmission term, we get

$$\bar{w}\Delta\bar{z} = cov(a, z_i) + cov(bz_p, z_i) - cov(cz_i, z_i) + cov(e_i, z_i). \qquad \text{(S1-3)}$$

The intercept (baseline fitness) is assumed to be the same for both altruists and selfish types, so its covariance is 0. Similarly, we assume the residuals do not covary with the trait, so $cov(e_i, z_i)$=0. Assuming 'b' and 'c' are constants (as we do in this model), with a bit of algebraic jiggery-pokery this becomes

$$\begin{aligned} \bar{w}\Delta\bar{z} &= b * cov(z_p, z_i) - c * cov(z_i, z_i) \\ &= b * var(z_i) * \beta(z_p, z_i) - c * var(z_i) \\ &= var(z_i)(b\beta(z_p, z_i) - c). \end{aligned} \qquad \text{(S1-4)}$$

We can interpret the regression coefficient '$\beta(z_p, z_i)$' as the coefficient of relatedness between partners ('*r*'), which is positive if, on average, interactants possess the same trait (i.e., altruists assort with other altruists). Assuming that variance is not 0 (by definition, variance cannot be negative), equation S1-4 simplifies to Hamilton's rule, where

$$\Delta\bar{z} > 0 \; if \; and \; only \; if \; rb - c > 0 \Leftrightarrow rB - C > 0 \qquad \text{(S1-5)}$$

It is important to note that in this example the costs (c) and benefits (b) to being altruistic in terms of game-theoretic pay-offs are equivalent to the direct fitness costs (*C*) and indirect fitness benefits (*B*) in Hamilton's rule. Other than in the simplest cases (like here) this will not hold. As such, it is important not to conflate the costs and benefits in pay-off matrices with direct and indirect fitness terms in Hamilton's rule (Gardner et al., 2011). For instance, in the first example in figure 2 in the main text cooperators pay a cost of 1, yet benefit from their own cooperation, so the direct fitness cost is negative (i.e., *C* = c - (b/2) = 1 - (3/2) = -0.5; -*C* is therefore positive, as - -0.5 = +0.5). A comparable approach to that presented above, but employing partial regression coefficients to calculate the '*B*' and '*C*' terms (a so-called 'generalised Hamilton's rule'), can be used to analyse such cases, including instances involving non-additive pay-offs, in terms of Hamilton's rule (Birch, 2014; Gardner et al., 2011; Marshall, 2015). However, the application of this regression-based approach is not without its critics, who argue that the regression approach can lead to model misspecification if fitness functions are non-linear (i.e., pay-offs are non-additive), as it applies a set of linear models to what may be a non-linear system (van Veelen, 2018; van Veelen, Allen, Hoffman, Simon, & Veller, 2017). It has also been argued that the regression method cannot make predictions, is unfalsifiable, and, in certain systems, can misclassify behaviours as 'cooperative' or 'costly' when they are not (Allen, Nowak, & Wilson, 2013; Nowak, McAvoy, Allen, & Wilson, 2017). This is not the place (and I am not the person) to adjudicate these technical debates, but for accessible and balanced reviews on this topic, see Birch (2017a; 2017b) and Okasha (2016). Regardless of these on-going debates, simple additive models – such as those presented above and in figure 2 in the main text – are a useful place to start for understanding these issues and how this kin selection perspective can be reformulated to a multi-level specification, to which we turn next.

Beginning with equation S1-1 we can also derive a multi-level selection equation for evolutionary change. Rather than take the global covariance between the trait and fitness for all individuals within the population, if the population is structed into groups we can decompose equation S1-1 into the covariance between group fitness – defined as the average fitness within said group – and the average trait in the group (denoted by capital letters with the subscript *k*) plus the average covariance between fitness and the trait within groups (denoted by lower-case letters with the subscript *jk*, meaning the *j*th individual in group *k*). Again assuming that the transmission term is zero, in this multi-level specification equation S1-1 now becomes

$$\bar{w}\Delta\bar{z} = cov(w_i, z_i) \Leftrightarrow cov(W_k, Z_k) + E(cov(w_{jk}, z_{jk})). \qquad \text{(S1-6)}$$

This is now a multi-level formulation of evolutionary change, where the first covariance term signifies the increased group-level fitness of groups with more altruists, while the second term is the average within-group covariance between within-group fitness and whether the individual is an altruist or not. These terms can be interpreted as reflecting the respective strengths of between-group selection and within-group selection (Sober & Wilson, 1998), although this interpretation has been contested (Okasha, 2006). Equation S1-6 can be made more intuitive by re-writing the covariance terms as variances and regression coefficients

$$\bar{w}\Delta\bar{z} = var(Z_k)\beta(W_k, Z_k) + E(var(z_{jk})\beta(w_{jk}, z_{jk})). \qquad \text{(S1-7)}$$

Thus, if the between-group regression coefficient is positive then altruists increase group fitness, while if the average within-group term is negative then altruists are selected against within groups. The relative magnitude of these regression coefficients and the levels of variation within and between groups will determine whether altruism spreads in the population or not. Given the assumptions of the model above, the between-group regression term will equal 'b-c', as this is the change in group fitness from a group with no altruists to a group with only altruists. The within-group regression term will equal '-(b+c)', as in mixed groups this is the relative loss in fitness for altruists compared to selfish types. Equation S1-7 then becomes

$$\bar{w}\Delta\bar{z} = var(Z_k)(b-c) - E(var(z_{jk})(b+c)). \qquad \text{(S1-8)}$$

This means that an altruistic trait which lowers within-group fitness can only evolve if it is outweighed by group-level selection. Therefore, with the Price equation as a starting point, both the kin selection and multi-level selection approaches can be seen as formally equivalent. Even though their end-points seem rather different, if constructed correctly both approaches lead to the same conclusions regarding the direction and magnitude of evolutionary change. This means that models of group selection can be rewritten into kin selection models, and vice versa (Marshall, 2011). For a more complex worked example, see the group selection model of Traulsen & Nowak (2006) which was rederived in terms of kin selection by Lehmann, Keller, West, & Roze (2007).

As a simple example of this equivalence between kin selection and MLS approaches, the 'scale of competition' influences cooperation between non-relatives and can be understood from both perspectives (West et al., 2006). If competition is 'local' (i.e., within groups), then lower levels of cooperation are expected. This can be understood from a kin selection perspective as cooperating with group-mates decreases one's direct fitness relative to non-cooperators in the group. Similarly, from an MLS perspective, if competition is local then there is no between-group selection, only within-group selection, so cooperation is not favoured as cooperators have lower fitness than defectors within groups. However, when competition is 'global' (i.e., between groups), from a kin selection view the costs to cooperating are lower, as cooperating with group-mates does not decrease one's relative fitness within-groups, but rather increases one's direct fitness when competing with other groups (as direct fitness now depends on group success). Alternatively, from an MLS perspective, global competition increases the level of between-group selection, thus favouring cooperative behaviour (as cooperative groups outcompete less-cooperative groups). Accordingly, experimental studies have shown that cooperation is greater when competition is between, rather than within, groups (Puurtinen & Mappes, 2009; West et al., 2006), while real-world studies suggest that conflict may promote cooperation, both in humans (Bauer, Blattman, Henrich, Miguel, & Mitts, 2016; although see Silva & Mace, 2015) and non-humans (e.g., green woodhoopoes: Radford, 2008).

A few further caveats are worth mentioning here. First, the MLS perspective requires bounded groups, where interactions only occur within these groups. Fluid or unbounded groups are an issue for the MLS framework as fitness cannot be attributed to groups (although a 'continuous trait-group' model involving overlapping groups can be formulated; Wilson, 1975). In contrast, a fluid population structure poses no problem for kin selection models (Birch, 2017b). Second, although the two approaches are mathematically equivalent (at least in simple additive models), it does not follow that they are causally equivalent. While both provide the 'correct' outcome of terms of overall evolutionary change, and in many cases both approaches are interchangeable, in some instances one approach provides the correct causal explanation while the other does not (Birch, 2019; Krupp, 2016; Okasha, 2016b). Third, although both approaches equivalently predict evolutionary change, they have different definitions of what qualifies as 'altruism' (see section 2 and figure 2 of the main text). Fourth, although the MLS perspective seemingly decomposes fitness into within-group and between-group selection, if groups of individually-fitter individuals cluster together, and fitness is determined by non-social factors, then this approach may erroneously detect that group selection is acting. This is despite group-level fitness effects being solely driven by individual-level differences in fitness that have nothing to do with the group (this is an example of causal non-equivalence, mentioned above). The canonical case of such 'fortuitous group benefits' is George Williams' (1966) deer example: A herd of fleet deer (an individual-level explanation) is not the same as a fleet herd of deer (a group-level explanation). As such, the multi-level Price equation may confuse a fleet deer for a fleet herd (for additional discussion of the alternative 'contextual analysis' approach to multi-level systems which may overcome some of these potential issues, see Okasha, 2006).

Finally, as mentioned above and in the main text, there is currently debate over the application of this regression-based approach to deriving Hamilton's rule when fitness pay-offs are non-additive (e.g., van Veelen et al., 2017), and therefore whether kin selection and MLS are in fact

mathematically equivalent under these circumstances (de Vladar & Szathmáry, 2017). Although all parties agree that kin selection and MLS are equivalent when fitness pay-offs are additive, critics of the equivalence thesis claim that under conditions of non-additivity Hamilton's rule is not uniquely defined, meaning that either the form of Hamilton's rule changes depending on the fitness pay-offs or population structure (van Veelen, 2011; van Veelen, García, Sabelis, & Egas, 2012; van Veelen, Luo, & Simon, 2014), or that use of the regression method opens the door to model misspecification if relationships are assumed to be linear when in fact they are non-linear (van Veelen et al., 2017). Additionally, although inclusive fitness is claimed to be the quantity that individuals are expected to maximise (Grafen, 2006), and therefore that natural selection will have designed organisms to act as if they possess the goal of maximising their inclusive fitness (West & Gardner, 2013), this conclusion only holds for additive models (Allen & Nowak, 2016; Nowak, Tarnita, & Wilson, 2010). In additive cases where the costs and benefits are under the actor's control and do not depend on population structure (i.e., regardless of the frequency of altruists in the population, if an individual acts altruistically they increase their recipient's fitness by a fixed amount '*B*', while decrease their own direct fitness by a fixed amount '*C*'), then organisms are expected to behave as if maximising their inclusive fitness. However, in non-additive situations the costs and benefits are not totally under the actor's control as these terms depend on the identity of their partner and the wider population structure (i.e., the benefit to others by acting altruistically (*B*) and the direct fitness cost to self (*C*) will vary depending on whether one's partner is an altruist or not, as well as the frequency of altruists in the population). In these circumstances, although Hamilton's rule will still be satisfied and a trait will be favoured via natural selection if $rB - C > 0$, organisms are not necessarily expected to act as if they are maximising their inclusive fitness as the direct and indirect fitness effects are, to some extent, beyond the actor's control (Birch, 2017a; Okasha, 2016a).

In response to these criticisms, proponents of kin selection and the equivalence thesis claim that the regression-based approach always correctly predicts the direction of selection, even in non-additive models, in both kin selection and MLS formulations, and therefore that both approaches are equivalent (Birch, 2014; Gardner et al., 2011; Marshall, 2015). This debate is far from settled, but to some extent this disagreement stems from different conceptions of Hamilton's rule and multiple definitions of the 'cost' and 'benefit' terms (Birch, 2014; Birch & Okasha, 2015; van Veelen et al., 2017). Additionally, given a potentially theoretically-reasonable assumption of 'weak selection' (where differences in fitness between altruists and non-altruists are small) non-additive models approximate additive models, removing some of the limitations associated with non-additivity, which also means that organisms may act as if maximising their inclusive fitness under these circumstances (Birch, 2017a; Okasha, 2016a). Furthermore, from a practical perspective for empiricists, the assumption that organisms possess the goal of maximizing their inclusive fitness can be a useful stance to take as it provides a 'design principle' from which to understand and interpret social behaviours without needing to know the underlying genetics of the population (Levin & Grafen, 2019; West & Gardner, 2013). Providing a detailed summary of these complex issues – let alone a resolution to them – is far beyond the scope of this paper, but it is hoped that this brief discussion provides an introductory sketch of these issues.

S2: Models of Cultural Multi-level Selection and Cultural Kin Selection

Genetic multi-level selection (equation S1-7 from section S1 above) can be adapted to represent multi-level cultural evolution (Henrich, 2004a):

$$\overline{wc}\Delta\bar{z}c = var(Zc_k)\beta(Wc_k, Zc_k) + E(var(zc_{jk})\beta(wc_{jk}, zc_{jk})). \quad \text{(S2-1)}$$

The additional 'c' letters indicate that equation S2-1 now denotes cultural fitness and cultural traits. Equation S2-1 states that the average change in a cultural trait from one cultural generation to the next ('$\Delta\bar{z}c$') is determined by: i) the group-level variation in the cultural trait multiplied by the slope between group cultural fitness and the average group trait (the between-group selection term); and ii) the average within-group variation in the cultural trait of each group multiplied by the slope between individual cultural fitness and possessing the cultural trait within each group (the within-group selection term).

CGS models make the evolution of group-beneficial behaviours more likely by either: i) increasing the slope of the between-group selection term, such as via group competition; ii) reducing the slope of the within-group selection term, causing the within-group slope to approach, equal or be greater than zero (meaning that within-group selection against cooperators is reduced or completely negated), such as via reciprocity, reputation, reward, punishment and other levelling mechanisms; iii) decreasing within-group variation and increasing between-group variation, by various social learning biases or by punishing non-normative behaviour; or iv) any combination of the above.

Given the equivalence between MLS and kin selection (uncontroversially in additive models, at least), equation S2-1 can be re-written as a form of cultural Hamilton's rule (Allison, 1992; Birch, 2017b; El Mouden, André, Morin, & Nettle, 2014), where the cultural trait can increase in frequency in the population when

$$r_c B_c - C_c > 0. \quad \text{(S2-2)}$$

The subscript 'c' denotes that equation S2-2 refers to cultural evolution, so B_c is the increase in recipient's cultural fitness, C_c is the direct cost to an individual's cultural fitness by performing said behaviour, and r_c is the cultural relatedness between the focal individual and partner(s). Cultural relatedness is the likelihood that interactants share the same culturally-inherited trait, beyond background levels of the trait in the population. As with genetic Hamilton's rule, a culturally costly behaviour can evolve if the cultural relatedness between interactants is high enough. For instance, an individual might relinquish some direct cultural fitness (C_c is positive), if it increases their indirect cultural fitness through others who share the same cultural trait ($r_c B_c > C_c$). Mechanisms of social learning, such as conformism and prestige bias, can cause cultural relatedness to be greater than genetic relatedness, while mechanisms such as punishment will reduce the direct benefit to acting selfishly. Although CGS is often not conceptualised in terms of a cultural Hamilton's rule, equations S2-1 and S2-2 are equivalent, meaning that cultural change can either be formulated as either a group-level process (equation S2-1) or an individual-level process (equation S2-2).

Note that these formulations are not measuring the impact of the cultural trait on *biological* fitness, but rather the impact of the cultural trait on *cultural* fitness, where cultural fitness is the amount of cultural influence that an individual or group has (El Mouden et al., 2014; Ramsey & De Block, 2017). Individuals (or groups) who spawn many cultural offspring have high cultural fitness (e.g., prestigious individuals or groups), while individuals (or groups) who have limited cultural influence have low cultural fitness (e.g., asocial individuals or groups with less-attractive cultural traits). This point is returned to in the main text (section 8). Note also that for simplicity equations S2-1 and S2-2 assume that transmission is unbiased, hence why the 'transmission' term (see Equation S1-1) has been omitted. Given that our evolved psychology and other sources of cultural learning are likely to bias culturally-inherited traits in certain directions, the transmission term for cultural evolution is likely to

be substantial compared to genetic evolution, but this additional complexity is ignored here (for additional discussion on this, see El Mouden et al., 2014).

References cited in supplementary information

Allen, B., & Nowak, M. A. (2016). There is no inclusive fitness at the level of the individual. *Current Opinion in Behavioral Sciences*, *12*, 122–128. https://doi.org/10.1016/j.cobeha.2016.10.002

Allen, B., Nowak, M. A., & Wilson, E. O. (2013). Limitations of inclusive fitness. *Proceedings of the National Academy of Sciences of the United States of America*, *110*(50), 20135–20139. https://doi.org/10.1073/pnas.1317588110

Allison, P. (1992). The cultural evolution of beneficent norms. *Social Forces*, *71*(2), 279–301. https://doi.org/https://doi.org/10.1093/sf/71.2.279

Bauer, M., Blattman, C., Henrich, J., Miguel, E., & Mitts, T. (2016). Can war foster cooperation? *Journal of Economic Perspectives*, *30*(3), 249–274. https://doi.org/https://doi.org/10.1257/jep.30.3.249

Birch, J. (2014). Hamilton's rule and its discontents. *British Journal for the Philosophy of Science*, *65*(2), 381–411. https://doi.org/10.1093/bjps/axt016

Birch, J. (2017a). The inclusive fitness controversy: Finding a way forward. *Royal Society Open Science*, *4*(7), 170335. https://doi.org/10.1098/rsos.170335

Birch, J. (2017b). *The Philosophy of Social Evolution*. Oxford: Oxford University Press.

Birch, J. (2019). Are kin and group selection rivals or friends? *Current Biology*, *29*(11), R433–R438. https://doi.org/10.1016/j.cub.2019.01.065

Birch, J., & Okasha, S. (2015). Kin selection and its critics. *BioScience*, *65*(1), 22–32. https://doi.org/10.1093/biosci/biu196

de Vladar, H. P., & Szathmáry, E. (2017). Beyond Hamilton's rule. *Science*, *356*(6337), 485–486. https://doi.org/https://doi.org/10.1126/science.aam6322

El Mouden, C., André, J. B., Morin, O., & Nettle, D. (2014). Cultural transmission and the evolution of human behaviour: A general approach based on the Price equation. *Journal of Evolutionary Biology*, *27*(2), 231–241. https://doi.org/10.1111/jeb.12296

Frank, S. A. (1997). The Price equation, Fisher's fundamental theorem, kin selection, and causal analysis. *Evolution*, *51*(6), 1712–1729. https://doi.org/https://doi.org/10.1111/j.1558-5646.1997.tb05096.x

Gardner, A., West, S. A., & Wild, G. (2011). The genetical theory of kin selection. *Journal of Evolutionary Biology*, *24*(5), 1020–1043. https://doi.org/10.1111/j.1420-9101.2011.02236.x

Grafen, A. (2006). Optimization of inclusive fitness. *Journal of Theoretical Biology*, *238*(3), 541–563. https://doi.org/10.1016/j.jtbi.2005.06.009

Henrich, J. (2004). Cultural group selection, coevolutionary processes and large-scale cooperation. *Journal of Economic Behavior and Organization*, *53*, 3–35. https://doi.org/10.1016/S0167-2681(03)00094-5

Krupp, D. B. (2016). Causality and the Levels of Selection. *Trends in Ecology and Evolution*, *31*(4), 255–257. https://doi.org/10.1016/j.tree.2016.01.008

Lehmann, L., Keller, L., West, S., & Roze, D. (2007). Group selection and kin selection: Two concepts but one process. *Proceedings of the National Academy of Sciences*, *104*(16), 6736–6739. https://doi.org/10.1073/pnas.0700662104

Levin, S. R., & Grafen, A. (2019). Inclusive fitness is an indispensable approximation for understanding organismal design. *Evolution*, *73*(6), 1066–1076. https://doi.org/10.1111/evo.13739

Marshall, J. A. R. (2011). Group selection and kin selection: Formally equivalent approaches. *Trends in Ecology and Evolution*, *26*(7), 325–332. https://doi.org/10.1016/j.tree.2011.04.008

Marshall, J. A. R. (2015). *Social Evolution and Inclusive Fitness Theory: An Introduction*. Princeton: Princeton University Press.

McElreath, R., & Boyd, R. (2007). *Mathematical Models of Social Evolution: A Guide for the Perplexed*. Chicago: University of Chicago Press.

Nowak, M. A., McAvoy, A., Allen, B., & Wilson, E. O. (2017). The general form of Hamilton's rule makes no predictions and cannot be tested empirically. *Proceedings of the National Academy of Sciences of the United States of America*, *114*(22), 5665–5670. https://doi.org/10.1073/pnas.1701805114

Nowak, M. A., Tarnita, C. E., & Wilson, E. O. (2010). The evolution of eusociality. *Nature*, *466*(7310), 1057–1062. https://doi.org/10.1038/nature09205

Okasha, S. (2006). *Evolution and the Levels of Selection*. Oxford: Oxford University Press.

Okasha, S. (2016a). On hamilton's rule and inclusive fitness theory with nonadditive payoffs. *Philosophy of Science*, *83*(5), 873–883. https://doi.org/10.1086/687871

Okasha, S. (2016b). The Relation between Kin and Multilevel Selection: An Approach Using Causal Graphs. *British Journal for the Philosophy of Science*, *67*(2), 435–470. https://doi.org/10.1093/bjps/axu047

Price, G. R. (1972). Extension of covariance selection mathematics. *Annals of Human Genetics*, *35*(4), 485–490. https://doi.org/10.1111/j.1469-1809.1957.tb01874.x

Puurtinen, M., & Mappes, T. (2009). Between-group competition and human cooperation. *Proceedings of the Royal Society B: Biological Sciences*, *276*(1655), 355–360. https://doi.org/10.1098/rspb.2008.1060

Radford, A. N. (2008). Duration and outcome of intergroup conflict influences intragroup affiliative behaviour. *Proceedings of the Royal Society B: Biological Sciences*, *275*(1653), 2787–2791. https://doi.org/10.1098/rspb.2008.0787

Ramsey, G., & De Block, A. (2017). Is cultural fitness hopelessly confused? *British Journal for the Philosophy of Science*, *68*(2), 305–328. https://doi.org/10.1093/bjps/axv047

Silva, A. S., & Mace, R. (2015). Inter-group conflict and cooperation: field experiments before, during and after sectarian riots in Northern Ireland. *Frontiers in Psychology*, *6*, 1790. https://doi.org/10.3389/fpsyg.2015.01790

Sober, E., & Wilson, D. S. (1998). *Unto Others: The Evolution and Psychology of Unselfish Behavior*.

Cambridge, MA: Harvard University Press.

Traulsen, A., & Nowak, M. A. (2006). Evolution of cooperation by multilevel selection. *Proceedings of the National Academy of Sciences of the United States of America*, *103*, 10951–10955. https://doi.org/https://doi.org/10.1073/pnas.0602530103

van Veelen, M. (2011). A rule is not a rule if it changes from case to case (a reply to Marshall's comment). *Journal of Theoretical Biology*, *270*(1), 189–195. https://doi.org/10.1016/j.jtbi.2010.11.011

van Veelen, M. (2018). Can hamilton's rule be violated? *eLife*, *7*, e41901. https://doi.org/10.7554/eLife.41901

van Veelen, M., Allen, B., Hoffman, M., Simon, B., & Veller, C. (2017). Hamilton's rule. *Journal of Theoretical Biology*, *414*(July 2016), 176–230. https://doi.org/10.1016/j.jtbi.2016.08.019

van Veelen, M., García, J., Sabelis, M. W., & Egas, M. (2012). Group selection and inclusive fitness are not equivalent; the Price equation vs. models and statistics. *Journal of Theoretical Biology*, *299*, 64–80. https://doi.org/10.1016/j.jtbi.2011.07.025

van Veelen, M., Luo, S., & Simon, B. (2014). A simple model of group selection that cannot be analyzed with inclusive fitness. *Journal of Theoretical Biology*, *360*, 279–289. https://doi.org/10.1016/j.jtbi.2014.07.004

West, S. A., & Gardner, A. (2013). Adaptation and Inclusive Fitness. *Current Biology*, *23*(13), R577–R584. https://doi.org/10.1016/j.cub.2013.05.031

West, S. A., Gardner, A., Shuker, D. M., Reynolds, T., Burton-Chellow, M., Sykes, E. M., … Griffin, A. S. (2006). Cooperation and the Scale of Competition in Humans. *Current Biology*, *16*(11), 1103–1106. https://doi.org/10.1016/j.cub.2006.03.069

Williams, G. C. (1966). *Adaptation and Natural Selection*. Princeton: Princeton University Press.

Wilson, D. S. (1975). A theory of group selection. *Proceedings of the National Academy of Sciences of the United States of America*, *72*, 143–146. https://doi.org/https://doi.org/10.1073/pnas.72.1.143
